# Crosstalk of the Brassinosteroid Signalosome with Phytohormonal and Stress Signaling Components Maintains a Balance between the Processes of Growth and Stress Tolerance

**DOI:** 10.3390/ijms19092675

**Published:** 2018-09-09

**Authors:** Damian Gruszka

**Affiliations:** Department of Genetics, Faculty of Biology and Environment Protection, University of Silesia, Jagiellonska 28, 40-032 Katowice, Poland; damian.gruszka@us.edu.pl; Tel.: +48-32-2009-482

**Keywords:** brassinosteroid, crosstalk, phytohormones, signaling, stress tolerance

## Abstract

Brassinosteroids (BRs) are a class of phytohormones, which regulate various processes during plant life cycle. Intensive studies conducted with genetic, physiological and molecular approaches allowed identification of various components participating in the BR signaling—from the ligand perception, through cytoplasmic signal transduction, up to the BR-dependent gene expression, which is regulated by transcription factors and chromatin modifying enzymes. The identification of new components of the BR signaling is an ongoing process, however an emerging view of the BR signalosome indicates that this process is interconnected at various stages with other metabolic pathways. The signaling crosstalk is mediated by the BR signaling proteins, which function as components of the transmembrane BR receptor, by a cytoplasmic kinase playing a role of the major negative regulator of the BR signaling, and by the transcription factors, which regulate the BR-dependent gene expression and form a complicated regulatory system. This molecular network of interdependencies allows a balance in homeostasis of various phytohormones to be maintained. Moreover, the components of the BR signalosome interact with factors regulating plant reactions to environmental cues and stress conditions. This intricate network of interactions enables a rapid adaptation of plant metabolism to constantly changing environmental conditions.

## 1. Introduction

Plants as sessile organisms, in order to adapt to the constantly changing environment and react to stress conditions, have evolved very effective molecular mechanisms, which allow rapid and adequate alteration in metabolic and physiological processes [1]. Regulation of these processes needs to proceed in a coordinated manner and is assured by a concerted action of various phytohormones [2]. Brassinosteroids (BRs) are polyhydroxylated steroidal plant hormones, which regulate a broad range of morphogenetic and physiological processes throughout plant life cycle [3,4]. It is known that the efficient regulation of various aspects of plant growth and physiological processes is achieved through a complicated network of interactions between components of the BR signaling and proteins participating in signal transduction pathways of other phytohormones [2,5]. This intricate molecular network includes also signaling pathways of environmental cues and stress signaling molecules (reactive oxygen species, ROS) [6,7].

Studies conducted for the last three decades with the genetic, biochemical, physiological and molecular approaches, carried out mostly in the model plant species *Arabidopsis thaliana* (thale cress), allowed various components of the BR signalosome to be identified and characterized functionally [8]. The numerous components of the BR signaling have been identified in Arabidopsis through application of various approaches, including chemical mutagenesis, activation tagging, T-DNA insertional mutagenesis, gene overexpression and RNAi-mediated gene silencing [4]. A complete signaling pathway that connects BR perception to the BR-dependent gene expression, which is mediated by nuclear transcription factors, has been uncovered [8,9]. Currently, the BR signaling is one of the best described molecular relays in plants [10,11,12,13]. A model of emergence and development of the BR signalosome during evolution of the plant kingdom has been recently proposed [14,15].

The BR ligand is perceived by the transmembrane receptor kinase Brassinosteroid-Insensitive1 (BRI1). The ligand perception triggers numerous phosphorylations in various domains of the receptor, which lead to activation of the kinase domain [8,16,17]. The BR molecule perception initiates conformational changes in the BRI1 structure, which enable interaction and transphosphorylations with four members of the Somatic Embryogenesis Receptor Kinase (SERKs) family [18,19]. Heterodimerization with the SERK kinases, and the BRI1-Associated receptor Kinase1/Somatic Embryogenesis Receptor-like Kinase3 (BAK1/SERK3) in particular, leads to the BR receptor complex formation and is required for full activation of the signaling pathway [20,21]. The formation of the activated receptor complex enables the BR signal transduction, which is mediated by a group of cytoplasmic proteins via a protein phosphorylation/dephosphorylation-dependent relay. The BR signal transduction between the activated, membrane-bound BRI1-SERKs receptor complex and the group of cytoplasmic regulators of the BR signaling is mediated by the BR-Signaling Kinases (BSKs), which belong to a subfamily of the Receptor-like Cytoplasmic Kinases [22]. Upon the BR perception, the BSK proteins are phosphorylated and activated by the BRI1 kinase, what results in dissociation of BSKs from the receptor complex [23,24]. Two homologous proteins—Constitutive Differential Growth1 (CDG1) and CDG-like1 (CDL1), belonging to a subfamily of cytoplasmic kinases, have also been identified as substrates of the BRI1 kinase and positive regulators of the BR signaling. Similar to the BSK proteins, the CDG proteins are phosphorylated and activated by the BRI1 kinase as a result of the BR perception [25]. The major role of the BSK and CDG proteins is interaction with and activation of the BRI1-Supressor1 (BSU1) phosphatase [26]. However, the BSK kinases function mostly as a scaffold during the BSU1 activation, whereas the CDG kinases are directly responsible for the stimulation of BSU1 activity through phosphorylation [8,22,25]. The BSU1 phosphatase plays a crucial role as a positive regulator of the BR signaling by repressing the activity of the Brassinosteroid-Insensitive2 (BIN2) kinase [26]. Upon activation by the BSK and CDG kinases, the BSU1 phosphatase interacts with and dephosphorylates the BIN2 kinase, which results in the BIN2 inactivation [22,26]. The BIN2 kinase plays a role of the major negative regulator of the BR signaling through phosphorylating and thus inhibiting two key transcription factors regulating the BR-dependent gene expression. BIN2 is encoded by a member of the subfamily of ten related genes—*Arabidopsis Shaggy-like Kinases* (*ASKs*). Several of the genes are redundantly involved in the BR signaling [27,28,29,30,31,32]. In the absence of BR, the BIN2 kinase autophosphorylates on Tyr-200 residue, what results in auto-activation [33]. The active BIN2 kinase phosphorylates and inactivates two closely related transcription factors—Brassinazole-Resistant1 (BZR1) and BRI1-EMS-Supressor1/Brassinazole-Resistant2 (BES1/BZR2), which are the major regulators of the BR-dependent gene expression [34,35]. The BIN2-mediated phosphorylation of the BZR1 and BES1 transcription factors reduces their DNA binding capacity, prevents them from dimerization with other transcription factors and leads to retention of the phosphorylated forms of BZR1 and BES1 in the cytoplasm [22,36,37,38]. However, the afore-mentioned kinases BSK1 and CDG1 may activate the cytoplasmic PP2A phosphatase in the BR perception-dependent manner, what eventually results in dephosphorylation and re-activation of the BZR1 and BES1 transcription factors [39]. Interestingly, it is known that the PP2A phosphatase is a dual-function regulator of the BR signaling, as apart from the positive influence on the BR signaling through the dephosphorylation and re-activation of the BZR1 and BES1 transcription factors, the PP2A phosphatase mediates a feedback regulation of the BR signaling by dephosphorylation of the active BRI1 receptor. The BZR1 and BES1 transcription factors constitute hubs of interactions with other transcription factors and histone modifying enzymes, which influence gene expression and form a complicated regulatory network of the BR-dependent gene expression [8]. BZR1 directly or indirectly regulates expression of about 80% of the genes, which are controlled by BRs through the BRI1 receptor. BZR1 inhibits expression of at least five BR biosynthetic genes and the *BRI1* gene, what constitutes a negative feedback mechanism [40]. BZR1 and BES1 regulate the BR-dependent expression of about five thousand genes, which are involved in various signaling pathways of phytohormones, environmental factors and stress conditions. Expression of about half of these genes is induced and the other half is repressed in the BR-dependent manner [41,42,43]. BZR1 and BES1 interact with some of their target-gene products, which function in the regulation of BR-dependent gene expression, in order to amplify the BR signaling [44,45]. Note that a group of the BR-regulated BZR1 and BES1 target genes encode at least 200 transcription factors, which modulate various BR responses in a secondary manner [43]. Thus, factors involved in regulation of the primary and secondary BR-dependent gene expression form a vast and complicated network of interactions and mutual dependencies, what enables a coordinated reaction of plant metabolism to constantly changing environmental conditions.

**Figure 1:**
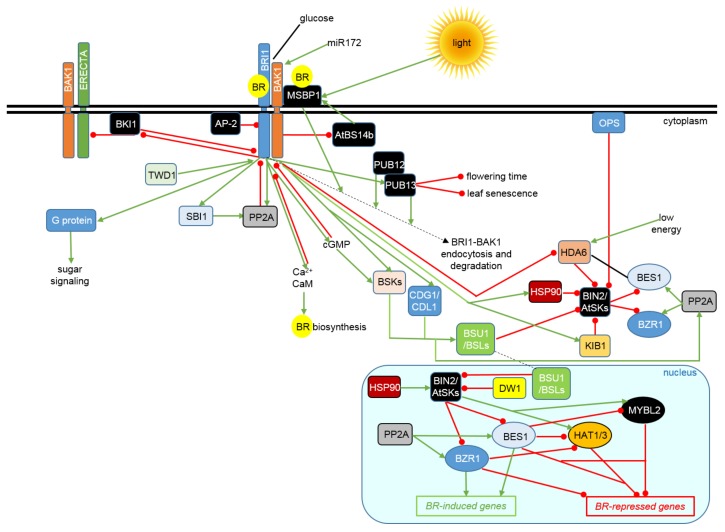
Model of the BR signaling regulation and control of the BR-dependent gene expression. Transcription factors are shown as ovals, other proteins are depicted as rectangles. Double black line represents the plasma membrane. Green arrows indicate stimulation, whereas red lines with the bullet points represent suppression. Detailed description is given in the text.

However, the identification of new components of the BR signaling is an ongoing process. The current model of the BR signaling regulation is depicted in Figure 1. An emerging view of the BR signalosome indicates that this process is interconnected at various stages with other metabolic pathways. This intricate network of interactions allows plant metabolism to adapt to the constantly changing environmental conditions. Moreover, the crosstalk of various signaling pathways enables a balance between the processes of growth and stress tolerance to be maintained. This review article presents a comprehensive and up-to-date description of the BR signalosome and its interactions with various signal transduction pathways, which allow the coordinated reaction to the environmental and stress conditions, as well as the maintenance of the balance between growth and stress tolerance.

## 2. The BR Signalosome—An Update

Expression of the *BRI1* gene is under developmental, organ-specific and diurnal regulation [46]. It is known that BRs attenuate expression of the *BRI1* gene [47], whereas auxin promotes transcription of the gene [48,49]. At low BR level some of the BRI1 receptors are present as dimers, what indicates that BRI1 may exist in the form of inactive dimers in the absence of BR. Moreover, some of the BRI1 receptors exist as constitutive dimers [50]. Interestingly, the BRI1-BAK1 hetero-oligomers may be partially preformed in the absence of BRs, therefore they function as a receptor complex able to perceive the ligand and initiate signaling, and the BR application significantly increases the BRI-SERK heterodimerization at the plasma membrane [51]. Interaction between the extracellular domains of the BRI1 and SERK proteins is the ligand-dependent and pH-dependent process [52,53], what suggests that the transmembrane domain and other cytoplasmic domains are required for the ligand-independent hetero-oligomerization [54]. Note that binding of the BAK1 extracellular domain does not induce any significant conformational changes in the extracellular domain of BRI1. However, the N-terminal part of the BAK1 Leucine-Rich Repeat (LRR) domain interacts with the BR molecule bound by BRI1, which results in the BR ligand incorporation between the two components of the receptor complex. Therefore, the assembly of the receptor complex is initiated by binding of the BR molecule by the BRI1 receptor, which is followed by the BAK1-mediated specific recognition of the newly BR ligand-formed surface via hydrophobic and hydrogen bonding interactions [53].

In the absence of BR, the BRI1 receptor is kept in its basal state by several mechanisms, which include auto-inhibitory effect of its C-terminal domain, autophosphorylation on Thr-872 in its kinase domain, and interaction with the BRI1 Kinase Inhibitor (BKI1) [55,56,57,58]. BKI1 contains an N-terminal motif, which anchors the protein in the plasma membrane and a C-terminal fragment, which interacts tightly with the BRI1 kinase domain preventing the association between the BRI1 and SERK kinases [17]. BKI1 competitively inhibits transphosphorylation on the cytosolic domains of BRI1 and BAK1 [59]. It was recently reported that association of the BKI1 protein with the plasma membrane occurs through electrostatic interactions with anionic phospholipids [60,61]. The BR perception by the ligand-binding domain of the BRI1 receptor causes conformational alterations [62,63], which allows the docking platform for the SERK proteins to be formed [52,53]. It is suggested that the BR molecule acts as a “molecular glue” which holds the BRI1 and SERK ectodomains together and allows close association of the C-terminal domains of the proteins [53,62]. The BR-dependent heterodimerization of the BRI1 and BAK1 ectodomains brings their cytoplasmic kinase domains in a proper orientation for transphosphorylation and competing with BKI1 [59]. The BR perception induces a rapid, BRI1-mediated phosphorylation on the conserved tyrosine residue of the BKI1 membrane-anchoring motif and an electrostatic switch, leading to a release of BKI1 from the plasma membrane and the BRI1 receptor, what allows the BRI1-SERK interaction, transphosphorylations within the receptor complex and signaling initiation [57,58,61]. It is known that the BKI1 and BSK proteins (the negative and positive regulator of the BR signaling, respectively) interact with different regions of BRI1 [59]. It was recently reported that BKI1 interacts also with another LRR Receptor-like kinase—ERECTA [64]. ERECTA and its homologs regulate various developmental processes, including stomatal patterning, petiole length, inflorescence elongation and ovule development [65,66,67]. Interestingly, BKI1 suppresses autophosphorylation of the ERECTA kinase and phosphorylation of its substrate—BAK1 [64]. BAK1 can act as a co-receptor of the BRI1 and ERECTA kinases [68]. BKI1 inhibits ERECTA kinase activity, and plays a role of negative regulator of the ERECTA signaling. Release of the BKI1-mediated repression of the ERECTA signaling depends largely on the BRI1 activation. It is suggested that the BR-induced dissociation of BKI1 from the plasma membrane (this process is independent of the ERECTA ligands) may attenuate the inhibitory effect of BKI1 on ERECTA, and eventually stimulate downstream ERECTA-mediated signaling. Hence, BKI1 acts as a common repressor of the BRI1 and ERECTA signaling processes (Figure 1). Note that both the ERECTA and BR signaling pathways enhance the *BKI1* gene expression by a feedback regulation [64].

The BR-activated signaling needs to be attenuated in a feedback mechanism to ensure hormonal homeostasis. The feedback regulation is mediated by the PP2A phosphatase, which dephosphorylates the activated BRI1 receptor [8]. A recent report indicated that cytoplasm-localized regulatory subunits (B’) of the PP2A phosphatase interact with and inactivate the BRI1 receptor through its dephosphorylation. BRs induce expression of these regulatory subunits, which interact preferentially with the phosphorylated form of BRI1. Thus, the rate of BR signaling is modulated by the PP2A-mediated and BR-dependent feedback inactivation of BRI1. Analysis of subcellular localization of the B’ subunits revealed that cytoplasmic PP2A dephosphorylates the BRI1 receptor and attenuates the BR response, whereas the nuclear pool of the PP2A phosphatase dephosphorylates the BZR1 transcription factor and consequently stimulates the BR response. Various subunits of the PP2A phosphatase have opposite effects on the BR signaling. Moreover, some of the subunits with the negative impact on the BR signaling play their role in a BIN2-dependent manner. Note that subcellular localization of the PP2A B’ subunits is a pivotal determinant of their dual role in the regulation of BR signaling, and relocating the subunits between the nucleus and the cytoplasm converted their role in this process. According to this model, BRI1 undergoes cycles of the ligand binding-induced phosphorylation-activation and the PP2A-dependent dephosphorylation-inactivation to ensure a rapid and efficient real-time monitoring of alterations in the BR concentration, which occur during plant development and in reaction to environmental cues [69]. It is known that the feedback-mediated regulation of the BRI1 activity occurs also through the BR-induced expression of the leucine carboxyl methyltransferase Suppressor of bri1 (SBI1), which methylates the PP2A subunits C leading to association of the PP2A phosphatase with the plasma membrane, what facilitates interaction with and dephosphorylation of the BRI1 receptor, and eventually its degradation [70] (Figure 1).

It was recently reported that the BRI1 receptor carries polyubiquitin chains on several lysine residues located in the cytoplasmic part of the protein. The polyubiquitin chains are organized through ubiquitin moieties linked by their lysine-63 residues and enhance a proteasome-independent BRI1 degradation. Expression of the non-ubiquitinatable, mutated version of the BRI1 receptor leads to an increase in the plasma membrane pool of the protein, and eventually to the BR hypersensitivity. Hence, it indicates that the cell surface is the primary site of the BR signaling initiation [71]. Activity of the BRI1 receptor may also be regulated in endocytosis-mediated manner. The BRI1 endocytosisis is dependent on the Adaptor protein complex-2 (AP-2), which is localized at the plasma membrane and interacts with clathrin. Interaction between AP-2 and BRI1 is independent on the presence or absence of BRs [72]. In fact, BR treatment does not alter the BRI1 endocytosis [73]. However, BR promotes a partitioning of BRI1 into functional membrane microdomains to stimulate the BR signaling. The membrane microdomains may function as platforms to increase the efficiency of interactions between the activated receptors and other signal transduction components, therefore the partitioning seems to be essential for the BR signaling [50]. Both BRI1 and BAK1 are distributed in the plasma membrane nanoclusters, which are largely immobile. Moreover, the ligand application does not change the number or composition of nanoclusters, but leads to a subsequent endocytosis after the signaling initiation. It was reported that the SERK co-receptors do not participate in maintaining the plasma membrane distribution of BRI1. The existence of nanoclusters could explain how one protein can play a role in various signaling pathways [54]. Interestingly, analysis of the *AP-2* gene mutants indicated that an impairment of the BRI1 endocytosis enhances the BR signaling. It indicated that the clathrin-mediated endocytosis is a negative regulator of the BR signaling [72,74] (Figure 1). It is postulated that some phosphorylated residues in BRI1 or particular phosphorylation patterns may influence the BRI1 endocytosis, apart from their participation in the initiation of BR signaling [72]. Several reports indicated that the BR signaling is mainly activated by the plasma membrane-localized BRI1. The BR signaling is stimulated when the BRI1 internalization is inhibited, whereas the BRI1 endocytosis is associated with the BR signaling attenuation [71,72,74]. It was recently reported that the BR signaling is positively regulated by the Brz-insensitive-long hypocotyl4 (BIL4) transmembrane protein, which is evolutionarily conserved in plants and animals. BIL4 is expressed at the early developmental stages and promotes cell elongation. BIL4 activates the BR signaling through interaction with the BRI1 receptor in endosomes. It is suggested that BIL4 inhibits BRI1 trafficking to vacuoles [75]. The internalization of BRI1 can be enhanced by ubiquitination [71]. A recent report indicated that endocytosis and protein abundance of the BRI1 receptor are regulated by the plant U-box (PUB) E3 ubiquitin ligase PUB12- and PUB13-mediated ubiquitination. It was shown that the BR perception enhances the BRI1 association with PUB12 and PUB13, what results in the BRI1 ubiquitination. BRI1 directly phosphorylates PUB13 on the serine-344 residue to promote its activity and association with the BRI1 receptor. Therefore, BRI1 is both the substrate and activator of PUB13. Interestingly, the BRI1 and BAK1 kinases are able to perform the PUB13 phosphorylation at different residues, what suggests that the differential phosphorylation of PUB13 may stimulate its interaction with different receptor kinases, and eventually leads to distinct signaling outputs. The PUB12- and PUB13-mediated ubiquitination is a crucial step in the BRI1 endocytosis and degradation [76] (Figure 1). Note that PUB12 and PUB13 were shown to influence the abscisic acid (ABA) signaling and chitin-induced immune responses by regulating the ubiquitination and protein abundance of the ABA and chitin receptor, respectively [77,78]. PUB13 also negatively regulates flowering time and leaf senescence in Arabidopsis [79,80]. It indicates that PUB13 plays various roles in controlling the phytohormone signaling, plant immunity and growth [76].

A recent report indicated that the BRI1 receptor kinase generates cyclic GMP (cGMP), what enables the cGMP-dependent signaling [81]. cGMP is an important signaling intermediate, which mediates various responses, such as alterations in ion transport, transcriptome and phosphoproteome [82,83,84,85]. The BRI1 kinase domain apart from the kinase activity shows a guanylate cyclase catalytic function. Moreover, the kinase activity is crucial for optimal cGMP production. Interestingly, cGMP inhibits the BRI1 kinase activity as a type of homeostasis control mechanism. Note that cGMP effectively stimulates phosphorylation of the above-mentioned BSK1 kinase. Hence, cGMP functions as a regulator that stimulates the BR signaling, but attenuates the signal generation by the BRI1 receptor [81] (Figure 1).

It was reported that the BR ligand perception by the receptor complex causes an immediate increase in the cytosolic Ca^2+^ concentration, what initiates the Ca^2+^ signaling cascade [86]. Calmodulin (CaM) proteins are sensors of the changes in the cytosolic Ca^2+^ concentration, which mediate cellular responses. Binding of Ca^2+^ to CaM induces its conformational changes, which enable interacting with target proteins resulting in alteration of their activities [87,88]. Interestingly, it was reported that Ca^2+^-dependent binding of the CaM protein to the kinase domain of BRI1 represses auto- and transphosphorylation activity of this kinase. It is suggested that the interaction between CaM and BRI1 constitutes a link, which connects the BR and Ca^2+^ signal transduction pathways [89]. Thus, the Ca^2+^-induced and CaM-mediated suppression of the BRI1 phosphorylation activity may ultimately attenuate the BR signaling in a negative feedback manner [86]. However, the interplay between the BR and Ca^2+^ signaling pathways appears to be more complex, as it was reported that one of the enzymes involved in the BR biosynthesis (DWARF1) is activated by CaM, what indicated that the cytosolic Ca^2+^ concentration may influence the BR homeostasis [90] (Figure 1).

Another report indicated that the BRI1 function is modulated by the Twisted Dwarf1 (TWD1) protein, which interacts with BRI1 in a BR-independent manner [91]. TWD1 is a protein belonging to an immunophilin family [92]. TWD1 positively regulates the BR-induced interaction between BRI1 and BAK1 and the BR-induced phosphorylation of these kinases. The interaction and phosphorylation between the BRI1 and BAK1 proteins are significantly reduced in the *twd1* mutant in response to the BR application, what suggest that TWD1 plays an important role during early steps of the BR signaling (Figure 1). However, the *twd1* mutation does not alter the BRI1 localization [91]. It is suggested that TWD1 facilitates proper folding and maturation of target proteins [93,94]. Therefore, TWD1 may play a role of a scaffold protein recruiting other protein (likely a chaperone) to proximity of BRI1 in order to enable an optimal folding of BRI1 [91]. Interestingly, it was reported that TWD1 localizes on the plasma membrane to regulate auxin transport [95].

The BR signaling is also regulated by a mechanism, which involves the AtBS14b protein belonging to the family of *N*-ethyl-maleimide sensitive factor adaptor protein receptor (SNAREs) domain-containing proteins. The SNARE proteins are known to participate in vesicle-associated membrane fusion. Overexpression of the AtBS14b caused insensitivity to exogenously applied BRs. AtBS14b directly interacts with the Membrane Steroid-Binding Protein1 (MSBP1) at vesicular compartments, but not with BRI1 or BAK1 [96]. The MSBP1 protein is localized in the plasma membrane and endosomes, and is able to bind the BR ligand. MSBP1 interacts with extracellular domain of BAK1 and promotes its endocytosis to negatively affect the BR signaling [97]. Interestingly, light inhibits the BR response by increasing the MSBP1 expression [98]. Transcription of the *AtBS14b* gene is down-regulated by BR in the BRI1-mediated process. AtBS14b acts via interacting with MSBP1 in the trans-Golgi network to enhance transport of MSBP1 to plasma membrane and its interaction with BAK1 [96] (Figure 1).

It was recently reported that physical interaction, phosphorylations between the BRI1 and BAK1 kinases and plasma membrane localization of the kinases are influenced by glucose in a concentration-dependent manner. Moreover, BRI1 and BAK1 directly interact with the G proteins, which are essential regulators of the sugar signaling (Figure 1). Interestingly, BRI1 and BAK1 phosphorylate subunits of the G protein, therefore it is suggested that BRI1 and BAK1 cooperate with the G protein subunits to regulate the sugar signaling and responses under light and dark conditions. Crosstalk between the sugar and hormone (BR) signaling pathways is essential for balancing carbon availability and plant development [99]. It is known that the BR and sugar signaling crosstalk proceeds through the hexokinase (HXK1) pathway to regulate hypocotyl elongation. Note that, glucose may regulate transcription level of 72% of the BR-regulated genes at the whole-genome level, 58% of the genes are regulated synergistically, whereas the rest of them were affected antagonistically. Moreover, glucose influences transcription of 80% of the BR-regulated transcription factor-encoding genes. BR and glucose co-regulate numerous genes involved in plant growth and in abiotic/biotic stress responses [100]. The recent report indicated that the BR and sugar signaling pathways may also converge at the G proteins to regulate different processes via different mechanisms [99].

It was also reported that the BR signaling is regulated through the miRNA-mediated mechanism. Overexpression of miR172 suppresses the *bak1* mutation effects, stimulates leaf elongation and increases root and hypocotyl growth during the seedling stage, what suggests that miR172 regulates vegetative growth by modulating the BR sensitivity [101]. It is known that an antagonistic action between miR172 and another miRNA (miR156) constitutes one of the best described examples of regulation of the developmental phase transition [102,103]. Expression of miRNAs may be up- or down-regulated by phytohormones. Moreover, more than one phytohormone may simultaneously regulate the same miRNA [101]. Hence, the miRNA-mediated mechanism may constitute another level of inter-hormonal crosstalk.

Recently, another mechanism of the BR signaling regulation has been described. The BSK kinases, apart from the kinase domain, contain a C-terminal tetratricopeptide repeat (TPR) domain, which functions as a phosphorylation-dependent autoregulatory domain to control the BSK activity. Function of the BSK proteins is conserved in Arabidopsis and rice (*Oryza sativa*) [11]. In Arabidopsis, the BSK protein family includes 12 members, which play partially redundant roles in the BR signaling. Each of the *BSK* genes displays a specific expression pattern at various stages of development. Moreover, BSKs compete among themselves for the interaction with BRI1, however they differ in their signaling capability [24]. The BRI1 receptor directly interacts with and phosphorylates the BSK proteins. The TPR domain directly interacts with the kinase domain within the BSK structure, and thus serves as the auto-inhibitory domain, which prevents BSK from precocious interacting with the BSU1 phosphatase when the BR signaling in attenuated under low BR concentration. The BRI1-mediated phosphorylation of BSK, which is induced by BR, disrupts the inter-domain association within the BSK protein, and eventually results in an enhanced interaction between the BSK kinase domain and the BSU1 phosphatase [11] (Figure 1). Interestingly, the BIN2 kinase interacts with the BSK proteins and phosphorylates them, however at different amino acid residues than the BRI1 receptor. This indicates a regulatory mechanism between the BSKs and BIN2 proteins, which plays a critical role in modulating the BR signaling. It was also suggested that the BSK proteins may function as homo- or heterodimers. The interaction between the BSK and BIN2 proteins validates the BSK function as a scaffold/docking platform, which facilitates interaction between the BSU1 phosphatase and BIN2, especially because BSKs lack conserved residues, which are crucial for kinase activity [24,25].

The BSU1 phosphatase belongs to a plant-specific protein family, which consists of four members (BSU1, BSL1, BSL2 and BSL3), which contain the Kelch-like repeat domains [26,104]. It was recently reported that additionally to their involvement in the BR signaling the BSU proteins show other specific and non-overlapping functions [105]. More recently, a subcellular localization of the BSU proteins was described. BSL1 shows a unique subcellular localization when compared with the other members of the BSU family. BSU1, BSL2 and BSL3 display both nuclear and cytoplasmic localization, whereas BSL1 is localized specifically in the cytoplasm [106]. The oligomeric combinations between the BSU1 proteins determine their subcellular localization. Interestingly, the oligomerization of the BSU proteins is not influenced by the BR signaling. However, nuclear localization of BSL1 enhances the BR signaling, what suggests that BSL1 plays a regulatory role in the BR signaling through retention of BSL2 and BSL3 in the cytoplasm. In the suggested model, nuclear localization of BSL2 and BSL3 is suppressed by oligomerization with BSL1, whereas cytoplasmic BSL1 is imported into the nucleus through association with BSU1. The BSU1-assisted translocation of BSL1 to the nucleus is probably caused by the fact that BSU1 accumulates in the nucleus more strongly that other members of the BSU family. Note that the cytoplasmic BSL1 forms homo-oligomeric complexes, whereas nuclear BSL1 occurs mainly in hetero-oligomers [107]. A detailed analysis indicated that a short, conserved amino acid sequence (KKVI) in the middle fragment of the BSU proteins is crucial for their oligomerization. Importantly, the BSU oligomers are more efficient in the BSK1 binding and the BIN2 Tyr-200 dephosphorylation in comparison with the BSU monomers. The BSU proteins form homo- and hetero-oligomers, which regulate their localization and are required for the effective BR signaling. It was suggested that the BSU proteins remain in a monomers-oligomers equilibrium irrespective of the BR signaling status, however upon the signaling initiation the upstream signaling components may preferentially interact with the BSU oligomers [106]. Since BSL1 is suggested to suppress the BR signaling by the cytoplasmic retention of BSL2 and BSL3, the BSL1-mediated oligomerization may constitute a negative feedback regulation of the BR signaling process [107].

The major negative regulator of the BR signaling, the BIN2 kinase, belongs to the family of Arabidopsis Shaggy/GSK3-like kinases (AtSKs), which consists of ten members. These kinases play diverse roles in the regulation of plant growth, stress responses, flowering and stomatal development [28,108,109]. Recent studies suggest that at least seven AtSKs are involved in the BR signaling [22,32]. One of the kinases, AtSK12 interacts with and phosphorylates BZR1, like BIN2 (AtSK21). Moreover, the AtSK12 protein level is regulated by BR and the BSU1 phosphatase, what indicates that AtSK12 together with BIN2 play the role of negative regulators of the BR signaling [22] (Figure 1). However, substrate specificities of the various BR signaling-related AtSKs and the proteins they interact with are still poorly described. Despite the similar function in the BR signaling regulation, the AtSK12 and BIN2 play diverse roles in the regulation of cell growth and stomatal development. AtSK12 regulates cell growth, but not stomatal development, whereas BIN2 regulates both these processes. It indicated that different AtSKs may have overlapping as well as specific functions [110].

Another mechanism of the BIN2 kinase activity regulation has been recently reported. Histone deacetylase HDA6 interacts with and deacetylates BIN2, what results in repression of its kinase activity. Therefore, HDA6 is a positive regulator of the BR signaling. Moreover, the amino acid residue, lys-189, was identified as the BIN2 acetylation site, which is deacetylated by HDA6 to impact the BIN2 activity. Acetylation neutralizes positive charge of the lys-189 side chain, which results in a decrease in phospho-binding by BIN2. Despite the direct interaction, BIN2 does not phosphorylate the HDA6 deacetylase. Interestingly, glucose may influence the BIN2 acetylation level, what suggests a regulatory connection to a cellular energy status. In this model, the acetylated form of BIN2 is more efficient in suppressing the BR signal transduction. Under the low energy conditions HDA6 is activated to deacetylate and thus inhibit the BIN2 kinase, what eventually results in the BR signaling stimulation. As a feedback mechanism, the BR signaling represses transcription of the *HDA6* gene. Surprisingly, a physical interaction between HDA6 and one of the major transcription factors, which regulate the BR-dependent gene expression, BES1, has also been reported, what suggests that HDA6 may modulate the BES1 activity, however a detailed mechanism is currently not known [111] (Figure 1). Recently, another mechanism of regulation of the BIN2 kinase has been described. The F-box protein KIB1 (*kink suppressed in bzr1-1D*), which is an E3 ubiquitin ligase, mediates a BR-induced ubiquitination and degradation of the BIN2 kinase, thus it is a positive regulator of the BR signaling. KIB1 directly interacts with BIN2 in a BR-dependent manner. The negative effect of KIB1 on BIN2 relies on dual mechanism, which involves preventing BIN2 from interaction with its substrate (BZR1) and subsequent ubiquitination of the BIN2 kinase, which results in its proteasomal degradation. As a consequence, it leads to accumulation of unphosphorylated, active form of BZR1. It is currently suggested that the BR-induced BIN2 inactivation proceeds in two steps: the BR signaling leads to an initial, partial inactivation of BIN2 via BSU1-mediated dephosphorylation, which is followed by the KIB1-mediated ubiquitination and ultimate degradation [112] (Figure 1).

The BIN2 kinase is also subject to another regulatory mechanism. The plasma membrane-associated protein OCTOPUS (OPS), which is specifically expressed in phloem [113], directly interacts with and suppresses the BIN2 kinase by sequestration at the plasma membrane, and consequently leads to accumulation of the unphosphorylated/active form of the BES1 transcription factor (Figure 1). The OPS-mediated recruitment of BIN2 to the plasma membrane prevents the inhibitory activity of the kinase in the nucleus. Hence, OPS is a positive regulator of the BR signaling pathway and promotes phloem differentiation [114]. In darkness the BIN2 activity is regulated by the CONSTITUTIVELY PHOTOMORPHOGENIC1 (COP1) ubiquitin ligase [115], whose function will be described in detail below.

Activity of the BIN2 kinase is also regulated by Heat Shock Protein 90 (HSP90) [116]. Heat Shock Protein90 is a crucial molecular chaperone present in high abundance in unstressed eukaryotic cells. HSP90 interacts with diverse proteins, representing various functional groups, regulating their folding and function [117]. Active HSP90 is required to retain BIN2 in the nucleus. Nuclear localization of HSP90 is inhibited by BR. Under control conditions (untreated cells) HSP90 interacts with BIN2 in the nucleus stimulating the BIN2 activity, what results in the inactivation of the BZR1 and BES1 transcription factors, whereas the BR application leads to interaction of these proteins in the cytoplasm, and eventually to BIN2 inactivation. It indicates that the site-specific action of HSP90 on BIN2 is BR-dependent. Thus, HSP90 plays a role of an integral component of the BIN2 activation mechanism [116]. Interestingly, an interaction of the HSP90 protein with another component of the BR signalosome, the BES1 transcription factor, was also reported [118,119]. Recently, in sorghum (*Sorghum bicolor*) a new component of the BR signaling has been identified. Protein product of the dwarfing gene (*DW1*) interacts with the BIN2 kinase and inhibits its nuclear localization, therefore DW1 functions as a positive regulator of the BR signaling. Comparative genomic analysis indicated that DW1 evolved in the land plant lineage after the divergence from chlorophyte. It is suggested that emergence of DW1 enabled plants to modulate the BR signaling through the BIN2 inactivation [15] (Figure 1).

Apart from the above-mentioned effect on the two major transcription factors which mediate the BR-dependent gene expression, BZR1 and BES1, BIN2 was reported to regulate stability and activity of many signaling components involved in the BR and other signaling pathways [108,120,121,122,123]. A gene encoding the Homeodomain-leucine zipper protein1 (HAT1) transcription factor was identified as a direct target gene of BES1, and the HAT1 transcription factor acts as the BES1 co-repressor. Function of the HAT1 and its homolog HAT3 (they act redundantly) is to stimulate the BR-mediated growth [124]. Expression of both homologs was found to be repressed by BR and regulated by BZR1 and BES1 [40,125]. HAT1 and BES1 interact with each other and bind to promoter sequences of some BR-repressed genes in order to synergistically suppress expression of the BR-repressed genes. Interestingly, HAT1 can be phosphorylated and (in contrast to most other BIN2 substrates) stabilized by the BIN2 kinase. HAT1 exists mostly in its phosphorylated form [124]. A similar mechanism has been described for another regulator of the BR-dependent gene expression. BES1 represses expression of the *Myeloblastosis family transcription factor-like2* (*MYBL2*) gene in a BR-dependent manner, and the MYBL2 protein, a transcription repressor, interacts with BES1 to co-operatively suppress the BR-inhibited gene expression. The inhibition of the BR-repressed gene expression is crucial for optimal BR response, therefore MYBL2 is a positive regulator of the BR signaling. However, BRs induce degradation of MYBL2 in a proteasome-mediated mechanism [126]. Both transcription factors, HAT1 and MYBL2, are substrates of BIN2, and the BIN2-mediated phosphorylation stabilizes these proteins, hence phosphorylation performed by the BIN2 kinase may have different effects on stabilities of the target proteins [124,126]. Therefore, it is suggested that BIN2 under specific conditions, may be a positive regulator of the BR signaling pathway, as it phosphorylates and stabilizes HAT1 and MYBL2, which function as positive regulators of the BR-dependent growth. HAT1 may be considered as an important regulator of the BR-repressed gene expression: when the BR signaling is attenuated the active BIN2 represses BES1 but stimulates the HAT1 function, whereas BRs promote the accumulation of active BES1 but inhibit HAT1 through transcriptional repression and lack of the BIN2-mediated stabilization [124] (Figure 1).

## 3. BES1 and BZR1—The Major Transcription Factors Regulating BR-Dependent Gene Expression form a Hub in the Network of Coordinated Gene Expression

**Figure 2:**
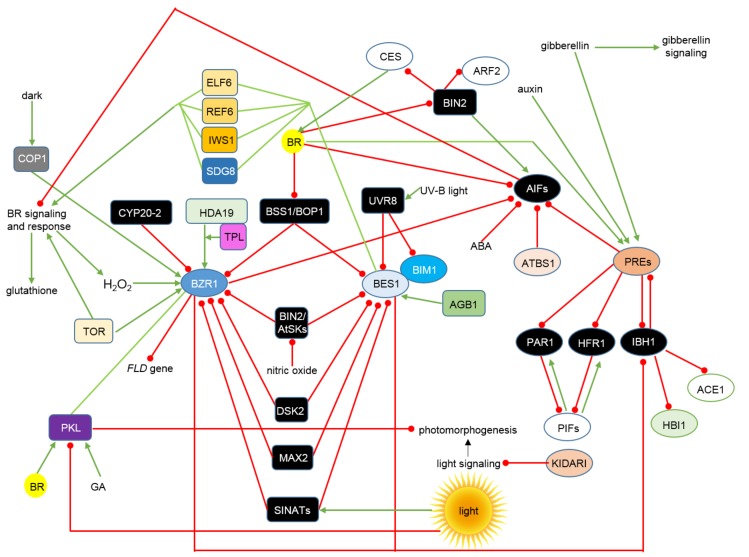
The complicated mechanism, which coordinately regulates the BR-dependent gene expression, with the BZR1 and BES1 transcription factors as hubs of the regulatory network. Transcription factors are shown as ovals, other proteins are depicted as rectangles. Green arrows indicate stimulation, whereas red lines with the bullet points represent suppression. Detailed description is given in the text.

The BZR1 and BES1 transcription factors constitute a core of the complicated network, which coordinately regulates the BR-dependent gene expression [8]. The complicated mechanism, which coordinately regulates the BR-dependent gene expression is shown in Figure 2. They are central factors that interact with a broad range of regulators and cofactors to modulate various developmental processes [127]. Moreover, recent discoveries indicated that these transcription factors represent an integration node of numerous signal transduction pathways, and this integration hub enables a coordinated regulation of various physiological processes in reaction to environmental and stress conditions [12]. An accumulating body of evidence provides new information about proteins interacting with and modulating activity of these transcription factors. It was reported that BZR1 directly interacts with and is phosphorylated by components of the Mitogen-Activated Protein Kinase (MAPK) complex, what indicates that BZR1 is post-translationally regulated by the MAPK pathway. Mechanism of the BZR1 regulation through phosphorylation may be quite complicated, as apart from 25 putative BIN2-mediated phosphorylation sites BZR1 is predicted to contain 11 phosphorylation sites for other kinases [127]. Phosphorylation status of BZR1 may also be regulated by other proteins. Interaction between the cyclophilin protein CYP20-2 and BZR1 stimulates the BZR1 phosphorylation and alters its conformation to repress its inhibitory transcriptional effect on the gene encoding a key regulator of flowering—*Flowering Locus D* (*FLD*) [128]. BZR1 interacts also with histone deacetylase HDA19 (HDA19), and this interaction is required for normal function of BZR1 and its role in regulating target gene expression [127]. Recently, a new component of the regulatory network has been identified. BZR-SENSITIVE-SHORT HYPOCOTYL1, also known as BLADE ON PETIOLE1 (BSS1/BOP1), is a negative regulator of the BR signaling. The *BSS1/BOP1* gene encodes a protein with ankyrin repeats, which regulates leaf development. The BSS1/BOP1 protein interacts with the BZR1 and BES1 transcription factors to form a cytosolic complex. Thus, the BSS1/BOP1 complex inhibits the BZR1 transport from the cytosol to the nucleus, and consequently negatively regulates the BR signaling. The protein complex is dissolved by the BR treatment, which enables the BZR1 transport to the nucleus. Moreover, the *BSS1/BOP1* gene expression is decreased by BR [129] (Figure 2).

It was recently reported that function of the BZR1 factor is positively regulated by hydrogen peroxide (H_2_O_2_), which oxidizes BZR1. The BR-activated and BRI1-mediated signaling stimulates accumulation of H_2_O_2_ to induce the BZR1 oxidation, and the H_2_O_2_ accumulation is required for the BR-dependent cell division and elongation. The H_2_O_2_-mediated oxidation results in enhanced transcriptional activity of BZR1 and promotes interaction of BZR1 with key regulators of auxin and light signaling pathways. The H_2_O_2_-dependent regulation affects expression of genes involved in several BR-related processes. Thus, BR and H_2_O_2_ control the BZR1 activity through distinct mechanisms, which involve nuclear localization/DNA binding and interaction with other factors, respectively [130]. Interplay between the redox homeostasis and the BR signaling may be quite complicated, as it is known that kinase activity of BIN2 is inhibited by nitric oxide in a dose-dependent manner [131]. Note that it has been recently reported in barley (*Hordeum vulgare*) that BR-insensitive mutants contain significantly lower concentrations of glutathione under the control conditions. Therefore, it was postulated that BR sensitivity is required for normal accumulation of this non-enzymatic antioxidant [132] (Figure 2).

It was recently reported that accumulation of BZR1 is regulated by sugar signaling, which is mediated by the Target of Rapamycin (TOR) kinase. TOR is an evolutionarily conserved regulator, which integrates nutrient and energy signaling to modulate plant growth. TOR plays a pivotal role in sugar-induced plant growth in the dark, and inhibition of TOR activity leads to plant growth repression and decreased expression of the BR-responsive genes. Thus, the TOR-mediated sugar signaling promotes plant growth through activation of the BR signaling and the BZR1 accumulation, whereas starvation induces the BZR1 protein degradation. This mechanism allows a balance between plant growth and carbon availability to be maintained [133].

Apart from the modifications of the BZR1/BES1 activity, phosphorylation status and the cytoplasm-nucleus shuttling, stability of these transcription factors is an important aspect in regulation of the BR signaling output. The recent years have witnessed a significant progress in elucidating the molecular mechanisms of the BZR1/BES1 stability regulation [12]. Proteins which are directly involved in the BZR1/BES1 degradation include the ubiquitin receptor protein Dominant Suppressor of KAR2 (DSK2) and three Skp-Cullin-F-box (SCF) E3 ubiquitin ligases—More Axillary Growth Locus2 (MAX2), Constitutively Photomorphogenic1 (COP1) and SINA of *Arabidopsis thaliana* (SINAT) [134,135,136,137]. The ubiquitin receptor protein DSK2 interacts with BZR1 and BES1 and targets these transcription factors to autophagy-mediated degradation during drought and starvation stress. This process involves an interaction between the DSK2 protein and the Autophagy-Related 8 (ATG8) protein, an ubiquitin-related polypeptide, which controls an autophagosome assembly and cargo recruitment [134]. The E3 ubiquitin ligases are conserved among eukaryotes, and in plants are known to play a role of central modulators of various phytohormonal signaling pathways. Moreover, about 100 proteins are involved in the hormone-mediated regulation of the E3 ligase activity [138]. The E3 ubiquitin ligase MAX2 is a key strigolactone (SL) signaling component, which inhibits plant shoot branching. MAX2 directly interacts with BZR1 and BES1, what results in degradation of these transcription factors. Thus, BRs and SL regulate the same developmental process by modulating the BZR1/BES1 stability [135]. COP1 and SINAT are involved in the light-regulated stability of BZR1, therefore these E3 ubiquitin ligases integrate the BR and light signalosomes. COP1 participates in the light signaling pathway, although it is a dark-activated enzyme, whose function is to capture and degrade the inactive/phosphorylated form of BZR1, what entails an increase in the ratio of the BZR1 active form, what results in plant growth stimulation [136]. The SINAT proteins interact with dephosphorylated forms of BZR1 and BES1 to mediate their ubiquitination and degradation. The abundance of SINAT proteins is stimulated by light, but decreased in the dark. Thus light/dark conditions regulate the BZR1/BES1 protein stability what enables a coordinated modulation of plant growth in the BZR1/BES1-dependent manner in response to the hormonal and environmental cues [137]. The SINAT-mediated ubiquitination of BES1 promotes the BES1-DSK2 interaction and subsequent BES1 degradation via selective autophagy [134,137]. Therefore, it becomes evident that subcellular localization, phosphorylation status, activity and stability of the BZR1 and BES1 transcription factors are dynamically and precisely regulated by multiple mechanisms [12]. It is suggested that the proteasome-mediated degradation and selective autophagy are involved in the BES1 inactivation, whereas SINATs ubiquitinate BES1 during starvation and light response. These multiple regulatory mechanisms of the BES1 accumulation enable integrating morphogenesis with various environmental cues, such as light or stress [138] (Figure 2).

Recently, a mechanism of molecular regulation of the BR signaling by the signal transduction pathway, which is mediated by the heterotrimeric guanine nucleotide-binding protein (G-protein), to cooperatively regulate cell division and elongation has been proposed [139]. G-proteins are crucial signal transducers composed of three subunits (Gα, Gβ and Gγ) which regulate diverse cellular processes [140]. G-proteins also affect multiple hormone responses to modulate plant developmental processes. The G-protein β subunit AGB1 directly interacts with the BES1 transcription factor and positively regulates the BR signaling through enhancing BES1 transcriptional activity. The interaction allows the BES1 target genes (including the BR biosynthesis genes *CPD* and *DWF4*) to be synergistically regulated. Moreover, the AGB1-BES1 interaction alters the BES1 phosphorylation status, which results in accumulation of dephosphorylated/active BES1 in the nucleus. Interestingly, AGB1 promotes proteasomal degradation of the phosphorylated form of BES1 [139]. AGB1 interacts also with the basic helix-loop-helix transcription factor BES-Interacting Myc-like (BIM1), which is known to form a heterodimer with BES1 and bind to promoters of the BR-responsive genes [141], what makes the role of the AGB1 protein in regulation of the BR signaling even more significant [139]. Another interaction that involves BES1 and BIM1 has been recently described. The interacting protein, UV Resistance Locus 8 (UVR8) is a UV-B light photoreceptor, which mediates the UV-B light response in plants [142]. The UVR8 proteins form homodimers in the absence of UV-B radiation, however exposure to UV-B promotes accumulation of the UVR8 monomers [143]. The UVR8 monomers interact directly with the COP1 E3 ubiquitin ligase to transduce the UV-B signal [144]. The UV-B light inhibits plant growth by repressing the BR response. This effect is underlain by the physical interaction of UVR8 with the active BES1 and BIM1 transcription factors to suppress their activities. The interaction of UVR8 with the BES1 and BIM1 transcription factors is UV-B-independent, however UV-B irradiation enhances the interaction. UVR8 is activated by the UV-B light and localized in the nucleus, where it represses the DNA-binding activities of BES1 and BIM1 to inhibit BES1-mediated transcription. Note that this mechanism of regulation of the BES1 and BIM1 activity represents also an important point of interplay between the BR and light signaling pathways. BES1 and BIM1 act downstream of UVR8 to regulate expression of genes related with cell elongation [142,143] (Figure 2).

The BR-dependent gene expression is regulated by numerous factors, which represent various types of transcription regulatory proteins. This complicated network of positive and negative interactions, which is coordinated by BZR1 and BES1 enables precisely controlled expression of various genes, which are related with the BR biosynthesis, the BR perception and signaling, as well as the BR-responsive genes [8]. BZR1, BES1, BES1-Homologs (BEH1-BEH4) and BES-Interacting Myc-like proteins (BIM1-3) are DNA-binding basic helix-loop-helix (bHLH) transcription factors, whereas Activation-tagged BRI1-Suppressor1 (ATBS1) and ATBS1-Interacting Factors (AIFs) are atypical non-DNA-binding bHLH proteins [145]. The bHLH proteins are highly conserved transcription factors regulating cell proliferation and differentiation. It is known that the bHLH proteins participate in various signaling processes, including the BR signal transduction. ATBS1 is an atypical bHLH protein, which is devoid of DNA-binding domain. Thus, function of ATBS1 as a positive regulator of the BR response relies on a heterodimerizing with and sequestering transcription factors, which play a negative role in the BR signaling [146]. Interestingly, ATBS1 shares a significant sequence similarity with KIDARI, which represses the light signaling [147] and Paclobutrazol Resistance1 (PRE1), which is involved in the gibberellin signal transduction [148]. ATBS1 interacts with and inhibits activity of four ATBS1-Interacting Factors (AIFs), which constitute a group of another atypical bHLH transcription factors [146]. AIFs function as negative regulators of the BR response and cell elongation [146,149] (Figure 2).

A similar mechanism has been described for another transcription factors participating in the BR signaling. The above-mentioned PRE1 protein and its rice homolog Increased Lamina Inclination1 (ILI1) may inactivate inhibitory bHLH transcription factors through heterodimerization [150]. Members of the PRE family of non-DNA-binding transcription factors promote plant growth through interacting with and sequestering another class of the HLH factors, such as PHYTOCHROME RAPIDLY REGULATED1 (PAR1), LONG HYPOCOTYL IN FAR-RED1 (HFR1), ILI1 binding bHLH1 (IBH1) and AIFs, which are all negative regulators of plant growth. PAR1 and HFR1 bind the PIF factors (which will be described below) and repress their DNA-binding activity, whereas PRE1 binds PAR1 and HFR1 to suppress their inhibitory effect on PIFs. Thus, the BR-mediated activation of PREs leads to suppression of the PAR1 and HFR1 activity, and ultimately to increase in the PIF activity [9,151]. Apart from the PRE1 activity, accumulation of the HFR1 and PAR1 proteins is also controlled by the COP1-mediated ubiquitination/degradation mechanism and through transcriptional activation, which is performed by PIFs [2]. BRs stimulate transcription of the *PRE1* and *ILI1* genes in the BZR1-dependent manner. The *PRE1* gene expression is also stimulated by auxin and gibberellin. PRE1 and ILI1 interact with the bHLH protein IBH1, which acts as a negative regulator of the BR response. Note that, BRs repress the *IBH1* gene transcription in the BZR1-dependent manner. BES1 also binds strongly to the *IBH1* promoter. Interestingly, the *PRE1* gene is expressed predominantly in young, growing tissues, whereas the *IBH1* gene is expressed mainly in mature organs, whose growth is arrested. PRE1 and IBH1 (as well as ILI1 and IBH1 in rice) antagonize each other at the protein level in regulating cell elongation. PRE1 (and ILI1) function as non-DNA binding HLH transcription factors that specifically suppress activity of IBH1. Therefore, BRs exert a double inhibitory effect on IBH1 at the transcript and protein level. This indicates that a conserved mechanism of BR-dependent regulation of plant development has evolved, which functions through the pairs of antagonizing HLH/bHLH transcription factors, which act downstream of BZR1. Moreover, members of the PRE1/ILI1 group of transcription factors integrate multiple signaling pathways, which regulate plant development [150]. The non-DNA-binding transcriptional repressor IBH1 interacts with the typical DNA-binding bHLH proteins Homolog of BEE2 Interacting with IBH1 (HBI1) and Activator of Cell Elongation1 (ACE1), what results in inhibiting their DNA-binding activities, and eventually in plant growth repression [152,153]. PRE1 prevents the IBH1 interactions with these transcriptional activators, thus maintains their DNA-binding activities and the growth-promoting effect. This network of interactions and dependencies forms a regulatory system of antagonistic mechanism, which regulates plant growth and development in response to multiple external and hormonal signals [145]. The tri-antagonistic bHLH network (ATBS1/PRE-AIFs/IBH1-HBI1/ACE1) functions to regulate plant growth in response to the BR, gibberellin, and light signaling and in the context of plant developmental stage. Components of the tri-antagonistic system can be either induced or repressed in the BZR1/BES1-dependent manner, what suggests a complex feedback regulation [43]. Another important module of the BR-regulated gene expression network has been recently described. The BR signaling-activated BZR1 directly represses transcription of the *ATBS1-Interacting Factor 2* (*AIF2*) gene. The *AIF2* gene encodes the atypical bHLH transcription factor, which has suppressive effect on plant growth and specifically interacts with and is phosphorylated by the BIN2 kinase [145]. Previously, it was reported that AIF1 also interacts with and is phosphorylated by the BIN2 kinase [146]. BRs induce dephosphorylation of AIF2 and the dephosphorylated form of AIF2 is directed to the proteasomal degradation. Apart from BRs, the AIF2 degradation is greatly stimulated by ABA, and to a relatively lower degree by other phytohormones. AIF2 constitutes an important regulator in modulation of the BR-dependent gene regulation, as the BIN2-mediated phosphorylation of AIF2 negatively regulates the BR response, whereas the BR-induced transcriptional repression and the AIF2 protein degradation enhance the BR signaling [145] (Figure 2).

Epigenetic modifications play important roles in gene expression regulation in response to various environmental stimuli and stresses. Recent reports indicated that phytohormones are also involved in the epigenetic modifications, which result in the altered gene expression [12]. The BR-regulated gene expression is also mediated by histone modifications, such as histone H3 Lys-27 (H3K27) demethylation and H3K36 methylations [43]. It is known that BES1 interacts with Early Flowering6 (ELF6) and Relative of Early Flowering6 (REF6), which play positive role in the BR signaling, regulating expression of about 1/3 of the BR-regulated genes. These interactions coordinate the BR-responsive developmental processes [154]. Both these factors contain a highly conserved Jumonji N/C domain characteristic for histone H3 demethylases [8]. REF6 is a H3K27 demethylase, which specifically demethylates the double- and triple-methylated H3K27 residue [155]. The H3K27 methylation is mediated by the Polycomb Repression Complex2 (PRC2) family of histone methyltransferases and it is known that double and triple methylation of H3K27 suppresses gene expression by recruiting the Like Heterochromatin Protein1 (LHP1) [156,157]. It is suggested that in response to BR, BES1 accumulates and recruits REF6 to target genes in order to remove the histone H3K27 di- and tri-methylation repression mark, what allows transcription to be activated [43]. Full transcriptional activity of BES1 requires association with the Interacting-With-Spt61 (IWS1) factor, which is known to participate in the transcription elongation process, RNA export and histone modifications and was found to be crucial for stimulation of the BR-induced gene expression [8,158]. IWS1 interacts with histone chaperon and another transcription elongation factor, Spt6, and possibly with other histone-modifying enzymes to regulate the BR-dependent gene expression. IWS1 plays a positive role in the BR-dependent gene expression, affecting 1/3 of the BR-induced genes. BES1 recruits IWS1 to promote transcription of target genes, what eventually results in the BR-induced gene expression [43] (Figure 2).

As far as the H3K36 methylation is concerned, it was reported that BES1 directly interacts with the SET Domain Group8 (SDG8) histone methyltransferase, which mediates the H3K36 di- and trimethylation, what enables modulating the BR-dependent gene expression [159]. A positive role of the H3K36 methylation in the BR response in rice was validated by identification of the H3K36 methyltransferase, which is encoded by the *SDG725* gene. A knockdown of the gene resulted in BR-related growth abnormalities of mutant plant. Moreover, expression of genes related with the BR biosynthesis, perception and signaling was down-regulated in the mutant. Importantly, levels of the H3K36 di- and tri-methylations were decreased in chromatin of the target genes, what indicated that the H3K36 methylation is an important, positive regulator of the BR-dependent gene expression [160]. However, the precise mechanism of the H3K36 methylation-dependent gene regulation is still not fully elucidated [43]. BZR1 interacts directly with the histone deacetylase HDA19, and a mutation of the *HDA19* gene negatively influenced expression of the BZR1-regulated target genes [127]. The interacting BZR1 and HDA19 proteins were reported to recruit another component—TOPLESS (TPL) to form a trimeric complex, which performs a coordinated regulation of cell elongation [161]. Interestingly, BZR1 interacts directly also with the chromatin-remodeling factor PICKLE (PKL), which represses photomorphogenesis. The PKL function is to remove the H3K27me3 marks from the target genes, what results in activation of genes related with plant growth [162] (Figure 2).

## 4. Interactions between the BRs Signalosome and Other Phytohormonal, Environmental and Stress Signaling Pathways Allow Maintenance of Physiological Homeostasis

**Figure 3:**
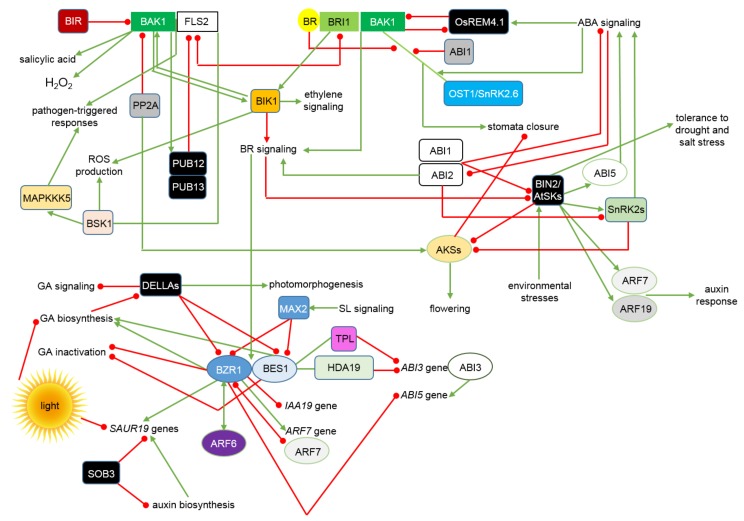
Crosstalk mechanism between the BR signalosome and the signaling pathways of other phytohormones. Transcription factors are shown as ovals, other proteins are depicted as rectangles. Green arrows indicate stimulation, whereas red lines with the bullet points represent suppression. Detailed description is given in the text.

It is known that BRs regulate the broad range of physiological processes during the plant life cycle through a complicated network of interactions and mutual dependencies with signalosomes of almost all other phytohormones [163]. Interactions between the BR signalosome and other signaling pathways involve proteins participating in various stages of the BR signal transduction relay: components of the transmembrane receptor complex, cytoplasmic proteins, which mediate the phosphorylation/dephosphorylation cascade, and members of the complicated network of transcription factors mediating the BR-dependent regulation of gene expression. The crosstalk mechanisms are depicted in Figure 3.

It was recently reported in rice that crosstalk between the BR and ABA signaling pathways may occur at the stage of the BRI1-BAK1 receptor complex formation. The crosstalk is mediated by the plasma membrane-anchored remorin proteins [164,165]. Transcription of the *OsREM4.1* gene, which encodes one of the remorin proteins, is promoted by the ABA signaling [165,166]. The OsREM4.1 protein binds to cytoplasmic domain of the OsSERK1 kinase (the BAK1 homolog), and this interaction prevents the BRI1-SERK1 complex formation, and consequently the BR signal transduction. The OsREM4.1 activity may be inhibited through a direct phosphorylation by the BR-activated BRI1 kinase [165]. The OsREM4.1 activity is stimulated by ABA to repress the BR-dependent physiological responses, whereas BRI1 inactivates the remorin in the BR-dependent manner. This underlies a trade-off mechanism which enables a balance between plant growth and stress responses, depending on the BR and ABA contents [167]. The BR-ABA interplay is also mediated by the BAK1 kinase in the process of regulation of stomata opening [168]. BAK1 interacts with Open Stomata1 (OST1)/Sucrose nonfermenting1-Related protein Kinase2.6 (SnRK2.6), which is the major regulator of the ABA-dependent stomata closure [169,170]. BRs negatively affect the BAK1-OST1 complex formation in the presence of ABA (Figure 3). BAK1 interacts also with the ABA-Insensitive1 (ABI1) protein, which belongs to the protein phosphatases 2C family. The BAK1 kinase and the ABI1 phosphatase regulate the OST1 phosphorylation in an opposite manner. Moreover, ABI1 interacts with BAK1 to prevent the BAK1-OST1 complex formation. It is suggested that formation of the BAK1-OST1 complex is enhanced by ABA and leads to stomata closure. On the contrary, this process is inhibited by BRs, what constitutes another aspect of the BR-ABA interplay and antagonism. Importantly, in the presence of both BR and ABA, BAK1 may act as a versatile component in signaling pathways of both phytohormones [168]. However, it was also recently reported that exogenously applied BR antagonizes the ABA-induced stomatal movements in a dose-dependent manner. BRs have also inhibitory effect on the ABA-induced ROS production [171].

It is known that the BR signaling antagonizes the pathogen-triggered responses [172,173]. Components of the BR receptor complex take also part in a molecular interplay between the BR signaling and plant immunity pathways. The BRI1 receptor kinase and the Flagellin-Sensing2 (FLS2) receptor, which binds bacterial peptides and subsequently initiates an antibacterial immune defense response, may compete for the common co-receptor BAK1. It results in the BR-mediated repression of the pathogen-triggered immunity [174,175]. The pivotal role of BAK1 in both BR signaling and pathogen-induced signal transduction indicates that BAK1 regulates the trade-off between plant growth and immune response [176]. In the absence of pathogens, the BAK1 activity may be repressed by different mechanisms, such as interaction with the regulatory BAK1-Interacting Receptor-like Kinase (BIR) proteins, which prevent initiation of the defense response [177,178]. Interestingly, BAK1 overexpression negatively affects plant development through stimulating immune responses in the absence of pathogens, as well as enhanced ethylene accumulation and the MAPK phosphorylation [179]. It was also recently reported that overexpression of the *BAK1* gene causes enhanced accumulation of salicylic acid (SA) and hydrogen peroxide, as well as increased expression of the cell death-regulating genes. Interestingly, the transgenic plant showed growth abnormalities despite of the enhanced BR sensitivity, due to occurrence of spontaneous cell death. Phenotype of the *BAK1*-overexpressing plant indicated that it results from a constitutive activation of the SA-regulated defense response [180]. It was also reported that subcellular localization of molecules involved in the endogenous SA accumulation is pivotal for the BAK1-mediated cell death regulation [1]. The Botrytis-Induced Kinase1 (BIK1) is a substrate of both the BRI1 receptor kinase and the FLS2-BAK1 transmembrane receptor, which requires induction of the MAPK pathway to trigger immunity response [167,174,175]. Pathogen infection and the FLS2-mediated ligand binding leads to the FLS2-BAK1 association, followed by BAK1-mediated phosphorylation and activation of BIK1, which dissociates from the complex. BIK1 transphosphorylates the FLS2-BAK1 receptor complex to fully activate the BAK1 kinase and transduce the signal, what leads to a burst in the ROS accumulation [167,181,182]. Interestingly, the FLS2 kinase activity is not required for the ligand-triggered FLS2-BAK1 interaction [183]. It should be noted that initiation and transduction of the pathogen-induced signaling needs to be tightly controlled, as it results in perturbations in plant growth and development [184,185]. Interestingly, the above-mentioned E3 ubiquitin ligases PUB12 and PUB13, which regulate the BRI1 endocytosis and degradation, directly ubiquitinate the FLS2 receptor and stimulate its degradation. PUB12 and PUB13 carry out the FLS2 ubiquitination after they are phosphorylated by BAK1, suggesting that BAK1 regulates the FLS2 degradation [186]. Activity of the BAK1 and BIK1 kinases is negatively regulated by phosphatases, however this process may be attenuated upon perception of the pathogen-associated ligand. Interestingly, the above-mentioned protein phosphatase PP2A, which is involved in the BR signal transduction, interacts with BAK1 and negatively regulates its activity [187,188]. BIK1 is known to interact with the kinase domain of the BRI1 receptor and is phosphorylated by this receptor in the BR-dependent manner. Interestingly, BRI1 directly phosphorylates BIK1 to regulate the BR signaling. Therefore, BIK1 participates in the pathogen-triggered signaling and in the BR signal transduction through distinct phosphorylation events by BAK1 and BRI1, respectively. BIK1 dissociates from the BRI1 and FLS2 receptors upon perception of the receptor-specific ligands. The ligand-induced release of BIK1 from the receptor complexes is associated with the BIK1 phosphorylation. BIK1 positively regulates the immune defense response, but functions as a negative regulator of the BR signaling. Therefore, BIK1 plays a role in maintenance of the plant growth versus immunity balance [176]. Note that it is known that BIK1 is also phosphorylated upon ethylene treatment and is required for a response to this hormone [189]. It was reported that BIK1 regulates the pathogen-triggered immunity by phosphorylating the NADPH oxidase, which catalyzes the ROS production [190,191]. Interestingly, the other cytoplasmic component of the BR signaling, BSK1, directly interacts with FLS2 and is required for the pathogen-induced burst in the ROS production, what indicates that BSK1 plays a positive role in the pathogen-triggered immunity [192,193]. It was recently reported that the cytoplasmic kinase BSK1 phosphorylates the Mitogen Activated Protein Kinase Kinase Kinase5 (MAPKKK5) to regulate plant defense responses [194]. It is known that BSK1 directly associates with the FLS2 receptor and mediates immune responses. Similar to BIK1, BSK1 binds to FLS2 in the absence of pathogen-derived ligand, but dissociates from the receptor upon the ligand perception to transduce the signal [192,195]. Activation of the MAPK relay is one of the initial steps during plant immune response [196]. The BSK1-mediated phosphorylation of MAPKKK5 is pivotal for the MAPKKK5 immune function in resistance to bacterial and fungal pathogens [194]. BSK1 serves also as a positive regulator of the FLS2-induced ROS production and accumulation of SA via physical interaction with FLS2. Inhibition of BSK1 increases susceptibility to pathogens [192,195] (Figure 3).

BIN2 is known to phosphorylate various transcription factors, including ARF2 [120], AIF1 [146], and CESTA (CES) [197] (Figure 2), which participate in the BR-dependent regulation of genes encoding proteins mediating crosstalk with auxin, cell elongation, and BR biosynthesis, respectively to regulate various aspects of plant development [111]. BIN2 is also known to participate in the BR-ABA signaling crosstalk, and constitutes a hub linking both BR and ABA signalosomes to coordinately regulate plant development and stress response [198]. BIN2 interacts with the SnRK2.2, SnRK2.3, and SnRK2.6 kinases and phosphorylates the first two. It is known that the BIN2-mediated phosphorylation is crucial for the SnRK2s’ activity as positive regulators of the ABA signaling, and thus enhances the ABA response [199]. The BIN2-mediated phosphorylation and activation of the SnRK2 proteins constitutes one of the mechanisms by which BIN2 stimulates the ABA responses [167]. The other mechanism relies on an interaction between BIN2 and one of the transcription factors participating in the ABA response pathway—ABA-Insensitive5 (ABI5). BIN2 phosphorylates ABI5 in an ABA-dependent manner, however it seems that sites of the BIN2-performed phosphorylation are different from those being phosphorylated by the SnRK2 kinases. Similarly to the positive effect of the BIN2-mediated phosphorylation on the SnRK2 proteins, it was reported that BIN2-dependent phosphorylation stabilizes the ABI5 transcription factor and is essential for the ABI5 function [200]. Note that several reports indicated that BIN2 and its homologs may be induced by environmental stresses [201,202,203]. Thus, the abiotic stress-activated BIN2 positively regulates the ABA response in the two ways: by phosphorylating and activating the SnRK2 proteins and by stabilizing the ABI5 transcription factor [167]. Moreover, several other ABA-regulated transcription factors were also reported to interact with BIN2 [200]. It is known that inhibition of the BR signaling by ABA is dependent on the ABI1 and ABI2 protein phosphatases 2C (negative regulators of the ABA signaling), which directly interact with BIN2 and dephosphorylate this kinase to attenuate its activity. ABA stimulates the BIN2 phosphorylation by inhibiting ABI2, which plays a positive role in regulation of the BR signaling. Since, as it was mentioned above, BIN2 interacts with and phosphorylates the SnRK2 kinases to activate them, this molecular interactions constitute a novel module PP2Cs-BIN2-SnRK2s in the ABA signaling pathway. The new mechanism shed a light on how plants maintain a balance between growth and stress response by coordinately regulating the respective signaling pathways. According to the current model of the BR-ABA crosstalk, under optimal growth conditions BRs promote plant growth and development by repressing BIN2, while the PP2Cs dephosphorylate SnRK2s to suppress the ABA response. PP2Cs may also dephosphorylate BIN2 to promote the BR signaling, but to repress the ABA response. Under stress conditions, ABA inhibits the PP2Cs’ activity to stimulate the SnRK2 and BIN2 kinases, what results in enhancing the ABA response and repressing the BR signaling [198] (Figure 3).

The BIN2 kinase and the PP2A phosphatase have recently been implicated in phosphorylation/dephosphorylation-mediated regulation of the ABA-responsive Kinase Substrates (AKS1, AKS2, and AKS3), which belong to bHLH transcription factors. It is known that ABA induces phosphorylation of the AKS transcription factors [204]. When unphosphorylated the AKS transcription factors promote stomatal opening [205]. It is known that apart from stomatal opening AKSs stimulate flowering. The AKS transcription factors are phosphorylated by BIN2, but dephosphorylated by the PP2A phosphatase, what suggests that this molecular interaction constitutes another example of the BR-ABA crosstalk. The ABA-induced AKSs phosphorylation is mediated by the SnRK2 kinases, whereas the BIN2-mediated phosphorylation of AKSs is inhibited by BRs. However, it is suggested that the ABA-induced phosphorylation of AKSs can be mediated by both BIN2 and SnRK2 kinases. The recent report indicated that BIN2 and PP2A regulate stomatal opening and flowering downstream of the BR and ABA response pathways [204]. BIN2 was also found to phosphorylate the auxin response factors ARF7 and ARF19 to suppress their interaction with the Aux/IAA repressor proteins, and consequently to activate these ARFs and enhance auxin response [206]. A recent study showed that overexpression of the soybean (*Glycine max*) *BIN2* gene promotes plant tolerance to drought and salt stress. It is known that expression of various representatives of the GSK3 family is stimulated by stress conditions. Interestingly, the soybean *BIN2* gene was overexpressed in Arabidopsis and led to the enhanced stress tolerance, indicating an evolutionarily conserved mechanism. Importantly, the *BIN2* overexpression caused up-regulation of the major drought-response gene, *RD29*, even in the absence of stress conditions [207] (Figure 3).

The BZR1 and BES1 transcription factors play a critical role in a crosstalk between the BR and gibberellic acid (GA) signaling pathways, and the BZR1/BES1-mediated inter-hormonal interplay occurs via direct protein-DNA and protein-protein interactions [208,209]. It is known that both these transcription factors bind to promoters of several GA metabolism-related genes to regulate their expression. In Arabidopsis, these factors bind in a BR-stimulated manner to promoter of the *GA20-oxidase 1* (*GA20ox1*) gene, which encodes an enzymes involved in the GA biosynthesis, to regulate its transcription [210]. In rice, BZR1 binds to promoters of the *GA20ox-2* and *GA3ox-2* genes, encoding the GA biosynthetic enzymes, and promoter of the *GA2ox-3* gene*,* which encodes an enzyme involved in the GA catabolism, to induce the GA biosynthesis but suppress the GA inactivation, what eventually results in stimulation of cell growth [211]. Apart from the control of the GA accumulation, the BZR1/BES1 transcription factors are known to directly interact with the DELLA proteins, which are the major negative regulators of the GA signal transduction [212,213,214]. GA-dependent stimulation of plant growth requires the BR-activated BZR1, as GA-regulated expression of more than thousand genes depends on BZR1 [212,213]. In response to the GA perception the DELLA proteins undergo the proteasome-mediated degradation, what results in a release of the growth suppression effect. The DELLA proteins are now known to participate as central regulators in various signaling pathways via direct protein-protein interactions [215]. DELLAs directly interact with the BZR1/BES1 transcription factors to inhibit their transcriptional activity and stability, what results in repressing cell elongation [214]. The GA metabolism is interconnected with photomorphogenesis, as it is known that the endogenous GA content is reduced by light and the subsequent accumulation of the DELLA proteins stimulates photomorphogenesis [216] (Figure 3).

Although synergistic physiological effects of auxin and BRs has been known for a long time, the underlying mechanisms have become elucidated only recently. BR and auxin have similar effect on expression of numerous genes, which are regulated synergistically or interdependently. An accumulated body of evidence indicated that an intact auxin signaling is crucial for the BR responses [12,48,120,217,218]. However, it should be kept in mind that the BR influence on auxin action is complex and may depend on developmental stage [42]. An important part of this inter-hormonal crosstalk involves activity of the BZR1/BES1 transcription factors. BZR1 regulates many genes involved in the auxin biosynthesis, transport and signal transduction. BZR1 binds to promoters of numerous auxin-responsive genes, and this transcriptional regulation relies on cooperation of BZR1 with Auxin Response Factors (ARFs) [40,42,125]. However, BZR1 may have a dual effect on expression of the auxin-related transcription factors: it attenuates transcription of the *IAA19* gene, but induces expression of the *ARF7* gene through direct binding to their promoters. Moreover, BZR1 interacts with proteins encoded by these genes to regulate transcription of a number of target genes, what enables a coordinated regulation of growth and developmental processes [219]. Note that the BZR1 and ARF7 transcription factors interact to co-regulate transcription of the *PHYB-4 Activation-Tagged Suppressor1* (*BAS1*) gene, which encodes an enzyme involved in the BR degradation. Interestingly, the BZR1 and ARF7 transcription factors compete with each other during binding to a common motif in the *BAS1* promoter and their interaction mutually decreases their DNA-binding capacity [220]. Another auxin response factor (ARF6) also interacts with BZR1 and this interaction mutually stimulates activity of these proteins in a synergistic regulation of transcription of common target genes [221]. Thus, BZR1 interacts with various auxin-related transcription factors through different modes and with various effects, what enables an integration of the BR and auxin signalosomes, and eventually the coordinated regulation of plant physiology [12]. It was also reported that the auxin signaling pathway acts as a nodal point, downstream of both BR and glucose signaling pathways, and is required for optimal plant growth during skotomorphogenesis. In the dark, glucose and BR act antagonistically at low glucose concentration, but synergistically at higher glucose concentration to regulate plant development [100]. It was recently reported that exogenous BR stimulates transcription of six genes, which encode members of the Small Auxin Up RNA19 (SAUR19) family, which promote cell elongation. Transcription of the *SAUR19* genes was enhanced in lines expressing a constitutively active form of BZR1 [222]. Experiments using the RNA-Seq technology also indicated that expression of the *SAUR19* genes is regulated by both GA and BR signaling pathways [45,212]. The *SAUR19* genes are repressed by Suppressor of Phytochrome B4-#3 (SOB3) and light, but were found to be induced by auxin. SOB3 is also known to repress expression of genes regulating the auxin biosynthesis [223]. Thus, SOB3 and the BR signaling converge to influence the *SAUR19* gene expression [222] (Figure 3).

In contrast to the above-described BR-GA and BR-auxin crosstalks, BRs and ABA are known for their antagonistic interplay during regulation of plant growth and development. The BZR1 and BES1 transcription factors take part in molecular mechanisms of this crosstalk. These transcription factors play their roles in this interplay through inhibiting expression of genes encoding the key components of the ABA signaling: ABA Insensitive3 (ABI3) and ABI5. BES1 forms a transcriptional repressor complex with the above-mentioned TPL transcription factor and Histone Deacetylase19 (HDA19), which mediates deacetylation of chromatin encompassing the *ABI3* gene. It results in repression of the *ABI3* gene transcription [224]. As ABI3 promotes expression of *ABI5*, the BES1-mediated suppression of the *ABI3* gene transcription results in down-regulation of both the ABA-related transcription factors, and consequently in attenuating the ABA responses [167]. Moreover, BZR1 binds to promoter of the *ABI5* gene and represses its expression, what results in a decrease in the ABA signaling [225]. Thus, BES1 and BZR1 may transcriptionally regulate the *ABI5* expression directly or through modulation of the *ABI3* gene expression, however a role of these two mechanisms remains to be elucidated [167] (Figure 3). The *MYB30* gene is one of the targets of the BES1 transcription factor and the encoded protein interacts with BES1 to regulate the BR-dependent gene expression [44]. MYB30 is also involved in regulation of the ABA response. Similar to BES1 and BZR1, MYB30 negatively regulates the ABA response. MYB30 is known to be sumoylated and stabilized by the Small Ubiquitin-like Modifier (SUMO) E3 ligase SIZ1 [226]. It is currently known that MYB30, being the negative regulator of the ABA response pathway, is controlled by various post-translational modifications which modulate its function [167]. BRs and ABA co-regulate expression of hundreds of genes. More than 35% of the BR-regulated genes are co-regulated by ABA [227,228].

The BZR1 and BES1 transcription factors were also reported to regulate the ethylene accumulation. Under low BR conditions, these transcription factors repress expression of the key genes involved in the ethylene biosynthesis by direct binding to their promoters [229]. However, it is postulated that at high BR concentrations ethylene biosynthesis is upregulated by stabilizing the key enzymes involved in this process, or indirectly through modifying the auxin-regulated ethylene accumulation [229,230].

Crosstalk between the BR signaling and the SL response also involves the BZR1/BES1 transcription factors. BZR1 and BES1 constantly interact with the MAX2 E3 ubiquitin ligase. The SL perception and signaling promote the MAX2-mediated degradation of the BZR1/BES1 transcription factors, what suppresses shoot branching [135]. Thus, it becomes evident that the BZR1/BES1 transcription factors constitute a hub in the complicated network of multi-signaling crosstalk, which enables a coordinated regulation of plant development and adaptation to environmental conditions (Figure 3).

**Figure 4:**
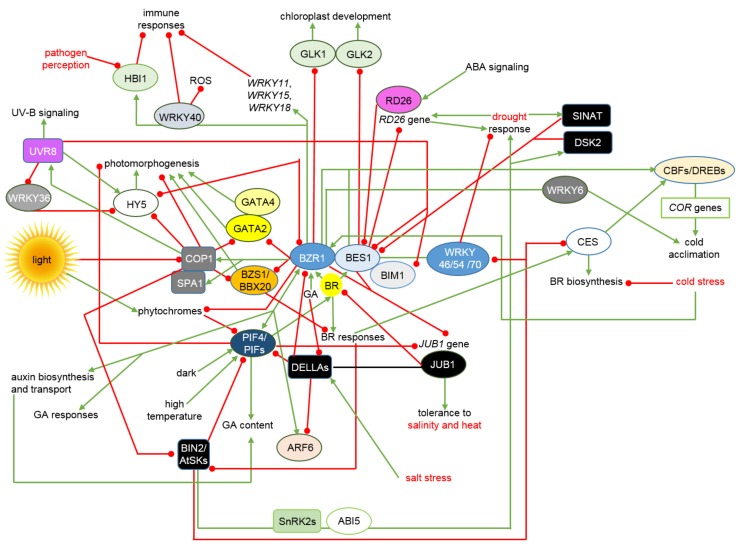
The BZR1 and BES1 transcription factors are hubs in the network of interactions with various environmental and stress signaling pathways. Transcription factors are shown as ovals, other proteins are depicted as rectangles. Green arrows indicate stimulation, whereas red lines with the bullet points represent suppression. Detailed description is given in the text.

It is known that BR and light co-regulate various aspects of plant growth and development [42]. In controlling photomorphogenesis, the BR signalosome is integrated with the light, gibberellin and auxin signaling pathways. Interaction between the BR and light signaling pathways involves also the BZR1/BES1 transcription factors, and their activity in this crosstalk may proceed through regulation of target gene transcription or via protein-protein interactions [12,42]. The crosstalk between the BR and light signaling pathways is regulated by a complex network of molecular interactions [231]. Interestingly, light does not significantly influence the endogenous BR concentration or the BR signaling upstream of BZR1 [42]. In the dark, BZR1 is activated by endogenous BRs and GA to stimulate growth [232]. BZR1 suppresses photomorphogenesis by direct binding to promoters of genes encoding 14 transcription factors mediating the light-regulated development [40,42]. In response to BR, BZR1 and BES1 suppress expression of two homologous genes encoding the GOLDEN2-LIKE (GLK1 and GLK2) transcription factors, which promote expression of numerous photosynthesis-related genes and are required for chloroplast development [40,125,233,234]. BZR1 binds to promoter of the *GATA2* gene, which encodes a transcription factor participating in the light signaling. GATA2 and GATA4 promote photomorphogenesis downstream of the BR and light signaling pathways through regulation of expression of numerous BR- and light-responsive genes. BZR1 has an inhibitory effect on the *GATA2* gene transcription and thus stimulates hypocotyl elongation [235]. However, GATA2 is post-translationally stimulated by light through inhibition of the COP1-mediated degradation [41,42]. Another positive regulator of photomorphogenesis, the B-box zinc finger BZS1/BBX20 transcription factor, is regulated in the similar manner—it is repressed by BZR1 at the transcriptional level, but activated by light at the protein level. BZS1 is a negative regulator of the BR responses. BZS1 is accumulated upon light exposure, however it is known that BZS1 may interact with COP1, what suggests that light stimulates the BZS1 accumulation through inhibiting the COP1-mediated ubiquitination and degradation. Hence, BZS1 regulates the crosstalk between BR and light signaling pathways [40,231] (Figure 4).

BZR1 participates also in thermomorphogenesis through direct binding to promoter of the *Phytochrome-Interacting Factor 4* (*PIF4*) gene, which encodes a transcription factor regulating this process [236]. The PIF4 accumulation is promoted by dark and high temperature [45]. BZR1 enhances the *PIF4* transcription in response to elevated ambient temperatures, what results in amplification of the signaling output [236]. Apart from the gene promoter-binding activity, BZR1 directly interacts with the PIF4 transcription factor, which is (like other PIFs) a negative regulator of photomorphogenesis [45,221,237,238]. Photoreceptors directly interact with and regulate accumulation of various transcription factors, including PIFs [239]. Active phytochromes inactivate PIFs by inhibiting their DNA-binding activities, and stimulating their phosphorylation, ubiquitination, and consequently degradation [238,240]. PIFs are important regulators of plant growth and responses the environmental cues [238]. Note that BZR1 represses expression of positive light-signaling components, such as photoreceptors, phototropin1, but stimulates expression of genes encoding negative regulators of photomorphogenesis, such the COP1 ubiquitin ligase and its interacting protein Suppressor of Phytochrome A1 (SPA1), which interact to mediate ubiquitination and degradation of the light-activated transcription factors [42,241,242]. The PIF4, BZR1, and BES1 transcription factors interact, what enables co-regulation of transcription of about 2,000 target genes, mostly in a synergistic manner [12,45,167]. BZR1 and PIF4 may bind to gene promoters as a heterodimer, or they may also act independently with other partners. Accumulation of PIF4 at dawn favors the PIF4-BZR1 interaction over BZR1 dimerization, thus de-represses the BR biosynthesis, and consequently stimulates plant growth [238]. Importantly, the GA signaling-related DELLA proteins suppress the DNA-binding activities of BZR1 and PIF4, what makes the BR, light and GA signaling pathways converge. BRs stimulate skotomorphogenesis by enhancing the nuclear accumulation of active BZR1. Gibberellin enhances etiolation through abolishing the inhibitory effect of DELLAs on BZR1 and PIF4. On the contrary, light promotes photomorphogenesis by inducing the PIF4 degradation. Note that the BZR1-PIF4 heterodimer directly activates transcription of genes involved in the auxin biosynthesis, transport, and the GA responses, but indirectly suppresses expression of genes, which encode chloroplast proteins. In the dark, accumulated PIFs increase the endogenous GA concentration, what results in degradation of the DELLA proteins, and promote expression of the *PRE* genes [42,216,238,243]. The light-induced PIF degradation entails a decrease in the GA content and the *PRE* expression, and consequently in an enhanced accumulation of DELLAs, what constitutes a core of a mechanism providing a switch between skoto- and photomorphogenesis [42]. The group of genes, which are co-regulated by the BZR1-PIF4 complex includes several members of the PRE family of transcription factors, which are known to stimulate plant growth downstream of the GA, BR and light signaling pathways [45,146,147,148,150]. Apart from light and GA, the DELLAs accumulation and activity are regulated by various hormonal and environmental factors, including ABA, auxin, cytokinin, ethylene, jasmonate, and stress conditions [244,245]. It is known that the DELLA-BZR1-PIFs module is a central hub of hormonal and environmental signaling crosstalk, which allows adjustment of plant growth and development to changing environmental conditions [42,195] (Figure 4).

Moreover, apart from the interaction with the PIF proteins, the BZR1/BES1 transcription factors interact also with the above-mentioned chromatin-remodeling factor PKL to form the PKL-PIF3-BZR1 complex, which promotes skotomorphogenesis by repressing trimethylation of the histone H3 Lys-27 on target gene promoters. The PKL protein accumulation is promoted by BR and GA application [162]. However, light represses PKL at transcriptional and translational level [246] (Figure 2). Note that the regulatory mechanism of coordinated gene expression is quite complicated, as it is known that activities of the BZR1/BES1, PIFs, ARF6, and the PKL proteins, which are all positive regulators of cell elongation, is suppressed through physical interaction with the DELLA proteins [12,162,212,221,247,248,249]. Thus, PKL acts as a point of integration of light, BR, and GA signaling pathways, which enables epigenetic regulation of plant development [162]. The BZR1, ARF6, and PIF4 transcription factors interact with each other and share numerous target genes [45,221]. These transcription factors mutually stimulate their DNA-binding and transcriptional activities, and consequently promote plant growth in an interdependent manner. This cooperative BZR1-ARF6-PIF4 interaction, and the inhibitory effect on their activity, which is exerted by DELLAs is termed as the BZR1-ARF6-PIF4/DELLA (BAP/D) module [2]. Action of the BAP/D module constitutes a hub in the crosstalk between the BR, auxin, PIF, and GA signalosomes, which regulates plant development in the dark [151]. Moreover, the BAP/D module allows various hormonal and environmental signaling pathways to converge and constitutes a control mechanism for coordinated regulation of plant growth and development [2]. Components of the BAP/D module may also act independently, regulating a subset of genes in a unique manner [45,221]. Interestingly, each of the components negatively regulates its own biosynthesis pathway, but promotes biosynthesis/signaling pathways of the partners [2]. It is known that BRs stimulate auxin transport, auxin enhances expression of some BR-biosynthetic genes, auxin and BRs increase the GA biosynthesis, and the PIF proteins promote the auxin and GA accumulations [151,238]. BIN2 phosphorylates and inactivates PIF4 and ARF2 (negative regulator of the auxin response), therefore the BR-mediated inactivation of BIN2 stimulates activities of these transcription factors [42,120,237]. Thus, BRs antagonize the light signaling by suppressing the BIN2-mediated degradation of PIF4. This mechanism allows timing hypocotyl elongation to late night, before the light signal and DELLA accumulation inhibit the PIF4 activity [237]. BZR1 directly interacts also with the basic leucine-zipper transcription factor Elongated Hypocotyl5 (HY5), which functions as a positive regulator of photomorphogenesis [250]. Theses transcription factors co-regulate expression of about 1170 target genes [40,251]. It is suggested that the BZR1-HY5 interaction is antagonistic in the regulation of target genes and enables a coordinated modulation of photomorphogenesis [12] (Figure 4).

The crosstalk between the BR and light signalosomes is also regulated by the above-mentioned E3 ubiquitin ligase COP1, which suppresses photomorphogenesis in the dark [136]. COP1 is a well-known repressor of photomorphogenesis, which mediates degradation of various transcription factors, which regulate light-responsive gene expression, in the dark [42]. As it was mentioned above, it was recently reported that COP1 and its interacting protein SPA1 may also negatively regulate activity of the BIN2 kinase in the dark, and thus prevent the BIN2-mediated phosphorylation and degradation of PIF3 [115]. The HY5, GATA2/4, and BZS1 transcription factors, which are all negative regulators of cell elongation are also degraded in the dark by COP1 [2]. COP1 is active in the dark, and light inhibits the COP1 activity through photoreceptor-mediated signaling to trigger various light responses [42,231]. On the contrary, COP1 is a positive regulator of the UV-B signaling and mediates the UV-B-induced nuclear accumulation of the UV Resistance Locus 8 (UVR8) photoreceptor [144,252,253]. COP1 together with UVR8 are required for the UV-B-induced transcription of the *HY5* gene [254]. Moreover, COP1 promotes the HY5 stability under the UV-B conditions [255]. It was reported that COP1 directly interacts with the phosphorylated form of BZR1 and causes its proteasomal degradation [136]. Other transcription factors mediating the BR response also participate in the BR-light crosstalk. It was recently reported that the SINAT E3 ligases mediate a direct ubiquitination and degradation of dephosphorylated form of BES1, hence SINATs negatively regulate the BR signaling. Interestingly, light stimulates the SINAT protein stability to regulate the BES1 accumulation. Although SINATs play the negative role in the BR signaling, the BR treatment does not influence expression of the *SINAT* genes or the encoded protein accumulation. The SINATs-BES1 interplay constitutes another point of crosstalk between the BR and light signalosomes, which regulate plant development. Note that degradation of the phosphorylated BES1 is very efficient in the dark, whereas the dephosphorylated form of BES1 is more vulnerable to degradation in the light. It is suggested that SINATs promote the degradation of dephosphorylated forms of BES1 and BZR1 under the light conditions, whereas COP1 degrades the phosphorylated forms of BES1/BZR1 in the dark [137]. BES1 and BES1-Interacting Myc-like 1 (BIM1) physically interact with the UV-B photoreceptor UVR8. This interaction impairs the DNA-binding activities of BES1 and BIM1 and consequently represses transcription of the BR-induced genes and hypocotyl elongation [142]. Recently, a model has been established, which suggests that in the absence of UV-B the UVR8 dimers are accumulated in the cytoplasm. In the nucleus, BES1 and BIM1 directly induce expression of the BR-responsive genes, whereas the WRKY36 transcription factor suppresses transcription of the *HY5* gene. The UV-B radiation promotes UVR8 monomerization and transport to the nucleus. As a consequence, UVR8 monomers interact with the BES1, BIM1, and the WRKY36 transcription factors to repress their activities, which results in stimulation of the *HY5* gene expression and inhibition of the BR-responsive gene expression [142,143,256] (Figure 4).

The BZR1 and BES1 transcription factors are also involved in a regulation of components, which mediate signaling pathways of various environmental stresses [4]. It was reported that about 17% of the BZR1 and BES1 target genes are involved in plant response to stress [257]. These molecular interactions underlie a crosstalk between the BR and stress signalosomes. It has been shown that BZR1, BES1, and PIF4 directly repress transcription of the *JUNGBRUNNEN1* (*JUB1*) gene, which delays senescence and stimulates plant tolerance to salinity and heat [258,259]. The JUB1 transcription factor plays a role of a negative regulator of the BR-dependent plant growth through suppressing the BR biosynthesis. This effect is caused by the fact that JUB1 interacts with DELLAs to suppress transcription of the BR and GA biosynthetic genes, including *DWARF4* and *GA3ox1* [260]. Therefore, it is suggested that BZR1/BES1 and JUB1 constitute a feedback loop, which enables a coordinated regulation and balance of plant growth and stress responses. Note that the JUB1 transcription factor is the major suppressor of plant growth, whose function is to transcriptionally attenuate the outputs of the BR, GA, and light signaling pathways [12]. It was reported that salinity stress activates the ABA signaling, but suppresses the BR and GA signaling pathways, what results in root growth arrest. The salt stress evokes a rapid accumulation of DELLAs, but leads to decrease in the nuclear accumulation of BZR1 [261] (Figure 4).

Phosphate deficiency results in a decrease in the BR biosynthesis and leads to cytoplasmic retention of the BZR1 and BES1 transcription factors in cells of the root elongation zone [262]. However, the low phosphate concentration in the environment causes an increase in the auxin content in primary root tip and lateral root primordia [263]. Taking into account the model of the BR-auxin antagonism in roots [264], the concurrent increase in the auxin content and decline in the BR accumulation lead to suppression of the primary root growth [2]. Moreover, the phosphate deficiency leads to decrease in the GA biosynthesis, what results in accumulation of the DELLA proteins [263]. Most probably, DELLAs also cause inhibition of root growth by modulating the BZR1 and ARF activities, however this mechanism requires further research [2].

Cold stress belongs to major environmental factors that adversely impact plant growth and development [257]. It was reported that BR treatment improves the plant cold tolerance [265,266,267]. Interestingly, transcription of the *CPD*, *DWF4*, and *BR6ox2* genes, which encode enzymes catalyzing the rate-limiting steps in the BR biosynthesis, was decreased in response to cold. The BR-regulated bHLH CESTA (CES) transcription factor, which positively regulates expression of the BR biosynthetic genes, contributes to a constitutive expression of C-Repeat Binding Factor/Dehydration-Responsive Element Binding Factors (CBFs/DREBs), which are key regulators of the Cold Responsive (COR) gene expression [268]. BRs control the CES protein activity, accumulation, and subcellular localization via BIN2-mediated phosphorylation and phosphorylation-repressed sumoylation, which is induced in response to the BR signaling [269]. Sumoylation promotes the CES activity in the cold tolerance. Thus, the CES transcription factor promotes basal freezing tolerance and the BR-dependent induction of the CBF gene expression [268]. The CBF/DREB transcription factors play a pivotal role in the cold acclimation. Overexpression of CBFs results in constitutively enhanced freezing tolerance [270,271,272,273,274]. It is suggested that BRs influence a basal *CBF* expression, but do not impact the *CBF* expression in early responses to BR or cold [268]. Cold stress induces accumulation of dephosphorylated/active form of BZR1. Moreover, gain-of-function mutations of the BZR1 and BES1 transcription factors led to an enhanced freezing tolerance. On the other hand, overexpression of the BIN2 kinase resulted in a decreased tolerance to cold stress. BZR1 and BES1 directly stimulate expression of the *CBF1* and *CBF2* genes, which encode transcription regulators playing the pivotal role in plant response to the cold stress. Moreover, BZR1 binds to promoters of several *COR* genes, whose transcription is regulated in the CBF-independent manner, to influence the cold stress response [257]. Thus, the CES, BZR1 and BES1 transcription factors positively regulate freezing tolerance via CBF-dependent and CBF-independent pathways [12,257,268]. It was also reported that one of the WRKY family of transcription factors (WRKY6) is a direct target of BZR1 and positively regulates freezing tolerance [257] (Figure 4).

Research conducted during the recent years provided an insight into the mechanism of crosstalk between the BR signalosome and molecular process of drought response. It seems that the BR response is suppressed during plant reaction to drought stress. The recent studies conducted in monocot and dicot species indicated that mutants with defects in the BR perception or biosynthesis show enhanced tolerance to drought [5,275,276]. The crosstalk mechanism is mainly relied on the BR-ABA antagonism. In contrast to the BIN2 kinase which promotes the drought response, the BZR1 and BES1 transcription factors play a crucial role in antagonizing the plant physiological reaction to drought. Under drought conditions, the active BIN2 phosphorylates and activates the SnRK2.2 and SnRK2.3 kinases and the ABI5 transcription factor [167]. The BZR1 and BES1 transcription factors also participate in the regulation of molecular interplay which occurs between the BR signalosome and drought signaling pathway. In response to BR, BES1 directly suppresses expression of the *Responsive to Desiccation26* (*RD26*) gene [277], which encodes a NAC transcription factor, and whose expression is stimulated by drought to promote tolerance against the stress conditions [278,279,280]. RD26 represses the BR-regulated plant growth under the stress conditions [167]. The RD26 transcription factor interacts with BES1 and represses its transcriptional activity (Figure 4). Therefore, the mutual inhibitory mechanism that occurs between the RD26 and BES1 transcription factors enables a coordinated regulation of balance between the processes of growth and drought response under changing environmental conditions [277]. Recently, a model has been suggested in which BRs suppress the drought responses under optimal growth conditions by inhibiting the *RD26* expression. Under the drought conditions, the *RD26* gene expression is rapidly stimulated and RD26 interacts with BES1 to repress its function [167]. It should be noted that during drought BES1 is ubiquitinated by the SINAT E3 ubiquitin ligases and targeted for degradation, which is mediated by the phospho-regulated ubiquitin receptor DSK2. DSK2 interacts with the ubiquitinated form of BES1 to carry out its selective autophagy. BIN2-mediated phosphorylation of DSK2 stimulates its interaction with the ATG8 protein, and eventually enhances the BES1 degradation [134]. The BES1 degradation during drought stress provides a key mechanism to suppress plant growth in favor of drought responses. Moreover, drought and the ABA signaling activate the RD26 transcription factor, which represses the BES1 activity, to stimulate expression of the drought-induced (BR-repressed genes) but suppress expression of the drought-repressed (BR-induced genes) [167]. It is also known that members of the WRKY (Trp-Arg-Lys-Tyr domain) family of transcription factors are involved in the regulation of balance between the BR-dependent growth and drought response. The WRKY46, WRKY54 and WRKY70 transcription factors are BR-induced and directly interact with BES1 to co-regulate expression of thousands of the BES1 target genes (including those involved in the BR biosynthesis and signaling). Activity of the WRKY factors is required for the BR-dependent growth. Importantly, the WRKY transcription factors are negative regulators of drought response. However, the WRKY factors are phosphorylated and destabilized by the BIN2 kinase. During drought, the BES1 and WRKY54 protein levels are significantly decreased. Interestingly, the triple *wrky46/wrky54/wrky70* mutant shows the BR-specific defects in growth but an enhanced drought tolerance, what confirms their dual role in regulation of the BR-dependent growth and drought response [281]. Moreover, activity of the BZR1 and BES1 transcription factors during drought is also regulated by the ubiquitin receptor protein DSK2, which directs these transcription factors to autophagy-mediated degradation under the drought and starvation stress conditions, as it was mentioned above [134]. However, it seems that BES1 is the major component of the BR-drought crosstalk, which may be inhibited in a triple mechanism under the stress conditions. The drought-induced BES1 suppression mechanisms include: the RD26-mediated repression of the BES1 transcriptional activity [277], the DSK2-mediated selective degradation [134], and destabilization of the WRKY transcription factors, which co-operate with BES1 in the BR-dependent gene expression regulation [281]. These mechanisms allow the BR-dependent growth to be attenuated in response to the drought stress. Thus, it is suggested that BZR1 and BES1 play a critical role in the regulation of the BR-drought crosstalk, which enables the balance between the plant growth and stress tolerance to be achieved and maintained [12]. It should be kept in mind that the BR-drought crosstalk is also regulated by the BIN2 kinase, which stimulates the ABA signaling components (SnRK2 and ABI5), but suppresses the BES1 activity directly or through DSK2-mediated degradation. It makes BIN2 the major component of the BR signalosome, which promotes the stress response, but inhibits plant growth [167] (Figure 4).

BRs act as multifaceted regulators of plant immunity, with divergent outcomes [195]. Apart from the involvement of the BZR1/BES1 transcription factors in the crosstalk between the BR signaling and the environmental cues and abiotic stresses, it is also known that these transcription factors participate in the interplay connecting the BR signalosome and mechanisms of plant immunity [12]. It was reported that the BR-induced activation of BZR1 causes suppression of plant immune signaling via several mechanisms [167]. This process is based on transcriptional regulation of the BZR1 target genes, but also on the protein-protein interactions. BZR1 promotes transcription of the several *WRKY* genes, *WRKY11*, *WRKY15*, and *WRKY18*, which encode transcription factors negatively regulating early immune responses. Moreover, BZR1 directly interacts with WRKY40 to repress expression of genes required for the immune response, and consequently to establish a balance between the BR signaling and plant immune response [282]. BZR1 directly interacts with WRKY40 to activate other WRKY factors in order to negatively regulate plant immunity [283]. It was reported that the WRKY40 involvement is crucial for the BR-mediated attenuation of pathogen-induced accumulation of ROS [282]. Moreover, the above-mentioned bHLH transcription factor HBI1, which is required for the BR-mediated cell elongation and enhancement of the BR-biosynthetic gene expression, represses transcription of crucial immune response genes. Therefore, HBI1 is one of the regulators, which maintain the balance between plant growth and immune response [284]. The BR signaling promotes expression of the *HBI1* gene and stability of the HBI1 protein [283]. On the other hand, expression of the *HBI1* gene is down-regulated in response to perception of pathogen-derived ligands [284,285]. It is suggested that BZR1 and HBI1 act as a regulatory hub at which multiple signaling pathways converge, enabling an effective modulation of the plant growth and immunity balance [283] (Figure 4). Surprisingly, it was reported that BES1 is a direct substrate of the Mitogen-Activated Protein Kinase6 (MPK6) in response to perception of pathogen-derived ligand. Moreover, the MPK6-mediated phosphorylation of BES1 is crucial for resistance against bacterial pathogens. Different residues of BES1 are phosphorylated in the BR and immune response pathways, what suggests differential modulation of the BES1 activity by the two signaling pathways. It indicates that BES1 plays a role of a positive regulator of the plant immune response, downstream of the MAPK signaling pathway. Positive role of BES1 in the plant immune response may be mediated by induction of the ROS production or through interaction with the MYB30 transcription factor [167,286]. Note that about 7% of genes which are regulated in the response to pathogen infection are the BES1-direct target genes, indicating an important role of BES1 in the pathogen-triggered signaling [286]. As mentioned above, the FLS2-initiated signaling leads to repression of the *HBI1* gene transcription, what results in growth inhibition and enhancement of plant immunity [284,285]. When overexpressed, HBI1 suppresses the immune response and the FLS2-induced growth inhibition, therefore HBI1 plays a key role in maintenance of the plant growth-immunity balance [2].

However, the relation between the BR signaling and the biotic stress-induced accumulation of ROS seems to be quite complicated, as it was reported that the H_2_O_2_-induced oxidative modification of the BZR1 and BES1 transcription factors stimulates their activity by promoting their interactions with the PIF4 an ARF6 transcription factors. The crosstalk between the BR signaling and plant immune response involves also another component. Thioredoxin protein, TRXh5, stimulates plant immunity, but suppresses plant growth. Transcription of the gene encoding TRXh5 is promoted by a pathogen infection, but negatively regulated by the BZR1/BES1 transcription factors. Moreover, TRXh5 may directly interact with BZR1 and BES1 to reduce their oxidation level and consequently the BR-related plant growth [130]. Therefore, the recently accumulated data indicated that BZR1 and BES1 form an important hub in the integration mechanism between the BR signalosome and pathogen-triggered signaling pathway, which enables maintaining the balance between the plant growth and biotic stress response [12]. It was reported that oxylipins, a group of oxygenated lipid derivatives, regulate plant development and immunity. Oxylipins activate the BR signaling to enhance cell wall-based defense to restrict pathogen infection. Interestingly, it was observed that the constitutive activation of the BR signaling in the *bzr1-1D* and *bes1-D* mutants caused increased resistance against bacterial and fungal pathogens. It is suggested that oxylipins require the functional BR biosynthesis and signaling to induce the cell wall-related defense responses and restrict pathogen infection. However, BRs alone are not sufficient to evoke this cell wall modification. It is suggested that oxylipins may act as ROS signals to activate the BR response and stimulate the cell wall modification and plant defense [287].

The above-described crosstalk mechanisms between the BR signalosome and the other phytohormonal and environmental signaling pathways enable the BR-dependent regulation of various developmental and physiological processes during the plant life cycle. Such interconnection of the diverse metabolic pathways allows the processes to be regulated in the coordinated manner. It is known that BRs promote seed development and germination and the post-germination plant growth through stimulating the cell division and elongation processes. Through interaction with other phytohormonal and light signaling pathways BRs are the key regulators of the physiological processes which occur during transition phase between skoto- and photomorphogenesis, which are critical for plant development. BRs also enhance expression of genes which encode α- and β-tubulin and regulate orientation of cortical microtubules, which influence arrangement of cellulose microfibrylls, and therefore BRs have impact on the cell wall structure. Moreover, BRs enhance differentiation of tracheary elements. It was also reported that BRs stimulate activity of transmembrane ATPases, what results in proton pumping to the apoplast and into vacuoles, and eventually in polarization of cell membranes. BRs were also found to increase the efficiency of photosynthesis by stimulating the Rubisco activity and consequently elevating the level of CO_2_ assimilation. BRs were also reported to stimulate leaf senescence. It was also found that exogenously applied BRs, especially at high concentrations, suppress root elongation but stimulate development of lateral roots. It was also reported that BRs have a stimulatory effect on plant reproductive development and regulation of flowering time. The recent years have witnessed further efforts aimed at elucidating the role of BRs in regulation of plant physiology, as many reports indicated that BRs may regulate the plant metabolic reactions to various biotic and abiotic stress conditions, including the pathogen-triggered reaction, drought and salt stress tolerance, thermotolerance, reaction to oxidative stress, as well as herbicide and pesticide tolerance [2,4,8]. The physiological reactions to these stress conditions are very complicated. As discussed in this review, the role of BRs in regulation of these processes based on involvement in the various crosstalk mechanisms is complex. Elucidating the role of BRs in these physiological processes has just begun and further research is needed to fully clarify these aspects.

## 5. BR Signaling in Cereal Crops—Novel and Specific Components

Semi-dwarf mutant cultivars of cereal crops are known to be more resistant to lodging, which is a cause of serious yield loss under unfavorable weather conditions. Therefore, application of the semi-dwarf cereal cultivars significantly contributed to a success of the so-called ‘Green Revolution’ in the second half of the twentieth century [288,289]. Moreover, the semi-dwarf, erect phenotype was shown to improve grain yield in high-density planting as it enables more efficient light capture [290,291,292,293]. BRs belong to principal growth promoting hormones involved in regulating wide spectrum of agronomic traits in cereals, such as plant height, lamina joint angle, tiller number and grain size. Thus, the BR signaling pathway is considered as an ideal biotechnological target to enhance crop yield and stress tolerance [266]. In fact, recent reports indicated that semi-dwarf mutants of rice and barley show prolonged tolerance to drought [5,294]. However, our knowledge about mechanism of the BR signaling in cereal crops is rather limited [295,296]. Therefore, identification and functional analysis of the BR-related genes in cereals is still very important, and mutation-based regulation of the BR-related growth reduction is still needed for an efficient breeding of cereal crops, especially taking into account the approaching global climate change. Transfer of information from the model (Arabidopsis) to cereal crop species is feasible, as mechanism of the BR signal transduction is evolutionarily conserved between various taxonomic groups of the plant kingdom.

Research conducted for the last two decades in rice enabled identification and functional analysis of genes encoding components of the BR receptor complex (OsBRI1 and OsBAK1), homologs of the BIN2 kinase (OsGSK1 and OsGSK2), the OsBZR1 transcription factor, proteins belonging to the 14-3-3 family, and a group of HLH transcription factors. Interestingly, the studies led to identification of some components of the BR signaling, which are specific for rice, such as the OsDLT, OsLIC and OsRAVL1 transcription factors, as well as the OsPRA2, OsELT1, OsTUD1 and OsGW5 proteins [4,297,298,299,300,301,302,303,304]. Interestingly, rice orthologs of the Arabidopsis proteins BSKs, BSU1, and PP2A have not been identified yet [304,305]. Homologous *BRI1* genes were identified and analyzed functionally in other cereal crops, such barley [289,306,307], purple false brome (*Brachypodium distachyon*) [308], maize (*Zea mays*) [309] and wheat (*Triticum aestivum*) [310]. Moreover, in wheat the *SERK* genes and two homologs of *BIN2* were characterized [311,312]. Nevertheless, among the monocots the BR signaling has been described to the greatest degree in rice.

A novel component of the BR signaling in rice has been recently identified. A small GTPase, OsPRA2, directly binds to the OsBRI1 receptor to inhibit its kinase activity. OsBRI1 and OsPRA2 co-localize at the plasma membrane. Thus, OsPRA2 is a negative regulator of the BR signaling [303]. Moreover, it was also recently reported that, apart from the inhibiting the OsBRI1 kinase activity, OsPRA2 represses its interaction with the OsBAK1 co-receptor to attenuate the BR response. Another protein which interacts with OsPRA2, the C2-domain containing GTPase Activating Protein1 (OsGAP1), stimulates the GTPase activity of OsPRA2, which is important for the regulation of the BR signaling [313].

It was recently reported in rice that the Enhanced Leaf inclination and Tiller number1 (ELT1) transmembrane receptor-like protein specifically interacts with the OsBRI1 receptor (the interaction occurs between the intracellular domains of the proteins), and promotes the BR signaling by repressing internalization and the endocytosis-mediated degradation of the polyubiquitinated OsBRI1. The ELT1-BRI1 interaction prevents the BRI1 ubiquitination. Thus, ELT1 acts as a positive regulator of the BR signal transduction by increasing the OsBRI1 receptor accumulation [296].

It was reported that in rice, apart from the OsBRI1 receptor, the Rice heterotrimeric G protein Alpha1 (RGA1) is also involved in the BR signaling [314,315]. In Arabidopsis, the orthologous G Protein Alpha1 (GPA1) is involved in the BR-mediated plant growth and the BR-dependent GA response during seed germination [316,317,318]. The rice RGA1 protein is also involved in the GA signaling [302]. However, a detailed relationship between the RGA1 function and the BR signaling remains not fully elucidated. It is suggested that RGA1 and OsBRI1 most likely function in parallel pathways. In contrast to OsBRI1, RGA1 is not involved in the feedback regulation of the BR biosynthesis [315]. Interestingly, it was suggested that the RGA1 protein may perceive auxin and other signals. Moreover, the RGA1 protein is involved in various developmental processes, such as germination, cell division and elongation, as well as plant morphogenesis. It was reported that, apart from the BR signaling, RGA1 mediates also the GA, pathogen-triggered, and the oxidative stress response pathways. RGA1 mediates a crosstalk between the BR and GA signaling pathways [299]. Recently, the barley *Brachytic1* gene, which encodes the alpha subunit of the heterotrimeric G protein has been identified and functionally analyzed [319,320].

It was reported that the RGA1 protein interacts directly with the Taihu Dwarf1 (TUD1) U-box E3 ubiquitin ligase (also known as Erect Leaf1, ELF1) and these proteins cooperate to mediate the BR signaling [302,305,321]. TUD1 localizes predominantly to the plasma membrane and acts as the BR signaling activator, hence the *tud1* mutant is insensitive to BRs. It is suggested that the TUD1 and RGA1 proteins directly interact to mediate the BR signaling pathway which is parallel or slightly overlapping with the canonical OsBRI1-mediated pathway. TUD1 functions directly downstream of RGA1 and mediates the G-protein signaling response to BR. Note that TUD1 is not involved in the GA signaling or biosynthesis and is not associated with the cytokinin metabolism [302].

Another component of the rice BR signalosome, DWARF and Low-Tillering (DLT) belongs to the plant-specific GRAS family of transcription factors. Expression of *DLT* is negatively regulated by exogenous and endogenous BRs in the OsBZR1-dependent feedback manner. However, DLT stimulates the *OsBZR1* expression, and this interaction enables fine-tuning of the BR response [297]. DLT is a positive regulator of the BR response, however it was found to be responsible for feedback repression of the BR-biosynthetic genes [299,300]. Interestingly, the DLT function is also inhibited by the OsGSK2 kinase (one of the two rice homologs of the Arabidopsis BIN2 kinase), which phosphorylates DLT. However, OsGSK2 phosphorylates OsBZR1 more preferably than DLT. BRs promote dephosphorylation of DLT [300,322]. Interestingly, both OsBZR1 and DLT phosphorylation and accumulation are developmentally regulated, as both transcription factors accumulate in their active, dephosphorylated forms in young plants [300].

Recently, a DLT-interacting protein, the Ovate Family Protein1 (OsOFP1) transcription factor was identified in rice, and found to be ubiquitously expressed. Expression of the *OsOFP1* gene is promoted by BRs in the OsBZR1-dependent manner. Note that, OsOFP1 belongs to a vast protein family and plays a redundant function with other OFP family members [323]. Interestingly, overexpression of the Arabidopsis homolog (AtOFP1) resulted in a dwarf stature caused by suppression of the GA biosynthesis [324]. Similarly, overexpression of the rice *OsOFP1* and *OsOFP2* genes also repressed the GA biosynthesis and resulted in a decreased plant height [323,325]. However, OsOFP1 plays a positive role in regulating the BR response to modulate rice plant architecture, and its function is dependent on DLT. Intriguingly, OsOFP1 is involved in the BR-induced repression of the GA biosynthesis, depending on the BR concentration or plant tissue. The OsOFP1 directly interacts with the OsGSK2 kinase. BRs promote the OsOFP1 protein stability by inhibiting the OsGSK2 kinase [323]. Another rice OFP protein, OsOFP8, also plays a positive role in the BR signaling and is stimulated by BR at transcriptional and protein level. Interestingly, the BR concentration that promotes the *OsOFP8* gene transcription inhibits the *OsBZR1* and *DLT* gene expression, what suggests a diverse regulatory mechanism. OsOFP8 specifically interacts with and is phosphorylated by the OsGSK2 kinase and the phosphorylation stimulates proteasomal degradation of OsOFP8 [326].

In rice the BR-GA signaling crosstalk is also mediated by the *OsMIR396d* gene. Overexpression of the gene results in an enhancement of the BR signaling, but in repression of the GA biosynthesis and signaling. OsBZR1 directly promotes accumulation of the OsmiR396d, which controls the BR response and the GA biosynthesis. The *OsMIR396d* gene expression is induced by applying a high concentration of GA. It indicates that OsmiR396d mediates the GA signaling and regulates the GA biosynthesis. Thus, it was suggested that OsmiR396d is involved in the feedback regulation of the GA biosynthesis [327].

Another factor, which is involved in the BR response in rice, Brassinosteroid Upregulated1 (BU1), belongs to a group of the HLH non-DNA binding transcription regulators [328]. BU1 shows high similarity with the Arabidopsis PRE1 protein. It was reported that expression of the BU1 gene is up-regulated by BRs, but repressed by ABA. Interestingly, BU1 is induced by BRs via both OsBRI1- and RGA1-mediated pathways [39,328]. It is suggested that function of the BU1 transcription regulator in the BR signaling relies on the HLH domain-mediated heterodimerization and repression of negative regulators of the BR response [299]. Most probably, OsBU1 acts downstream of the OsBRI1- and RGA1-mediated BR response pathways [328].

Another HLH protein in rice, Increased Leaf Inclination1 (ILI1) shares significant similarity with the above-described BU1 from rice and PRE1 from Arabidopsis. Function of the ILI1/PRE1 proteins is conserved in monocots and dicots. Expression of the *PRE1* gene is positively regulated by BRs, auxin and GA, therefore PRE1 may regulate the interplay between these hormones. ILI1 interacts with the bHLH transcription factor ILI1-binding bHLH protein1 (IBH1). Expression of the *IBH1* gene is down-regulated by BRs. As mentioned above, function of the ILI1 protein is similar to the PRE1 activity in Arabidopsis—the BR-induced HLH proteins (ILI1 and PRE1) interact with and inactivate the negative regulators of the BR response, the bHLH transcription factors IBH1. The negative regulation of the *IBH1* gene expression is also conserved in these species—BRs suppress its transcription in the BZR1-dependent manner. Thus, BRs inhibits the IBH1 function at the transcriptional and protein level [150]. Therefore, the regulatory mechanism of the HLH-bHLH antagonistic interactions between transcription factors ILI1 and IBH1 (in rice) and PRE1 and IBH1 (in Arabidopsis) is evolutionarily conserved [299].

Another rice-specific transcription factor, Leaf and Tiller Angle Increased Controller (OsLIC), functions antagonistically to OsBZR1 in the BR response. OsLIC acts as a negative regulator of the BR signaling and represses the BZR1 activity by attenuating transcription of the *OsBZR1* and *ILI1* genes. Moreover, OsLIC activates transcription of the *IBH1* gene [301]. On the other hand, OsBZR1 represses transcription of the *OsLIC* and *IBH1* genes, but stimulates transcription of the *ILI1* gene, thus antagonizes the OsLIC function [150,301]. Intriguingly, functions of the OsBZR1 and OsLIC transcription factors are repressed by the OsGSK1 kinase [322].

Another component of the BR signalosome in rice, Related to ABI3/VP1 RAV-Like1 (RAVL1), is a B3-domain transcription factor that regulates the BR homeostasis by directly activating expression of the *OsBRI1* and the BR biosynthetic genes. Interestingly, in contrast to OsBZR1, RAVL1 is not involved in feedback repression of these genes, and therefore it is suggested to ensure a basal activity of the BR metabolism [298]. However, the RAVL1 activity is not modulated by BRs, what points to a potential interplay between BRs and other hormones via RAVL1. Indeed, it was reported that 2,4-D (a synthetic auxin) treatment induced, whereas the 1-aminocyclopropane-1-carboxylic acid (ACC, ethylene precursor) application suppressed the *RAVL1* expression level. The other hormones do not seem to alter the *RAVL1* expression. It is suggested that auxin response factors (ARFs) might be involved in the auxin induction of *RAVL1* transcription, however further studies are required. RAVL1 activates both BR and ethylene signaling in rice. RAVL1 was also found to activate ethylene-related genes and positively regulate the ethylene response. Thus, suppression of the *RAVL1* expression by ethylene might be a part of feedback mechanism. Interestingly, RAVL1 may activate the ethylene response in the BR signaling-independent manner. Moreover, RAVL1 is suggested to activate the *OsBRI1* and the BR-biosynthetic genes independently of the ethylene signaling [329].

Recently, a novel regulator of the BR signaling in rice has been identified. The *GW5* gene is expressed in various rice organs and encodes a calmodulin-binding protein. The GW5 protein is localized to the plasma membrane and directly interacts with and represses the OsGSK2 kinase. GW5 inhibits both auto- and transphosphorylation activity of OsGSK2 [304]. It is known that GSK2 phosphorylates the OsBZR1 and DLT transcription factors, what results in suppressing their activities and eventually in proteasomal degradation [300,305]. The GW5 activity results in accumulation of active transcription factors OsBZR1 and DLT, and consequently the enhanced BR-responsive gene expression and growth responses. Thus, GW5 is a positive regulator of the BR signaling in rice [304].

It was recently reported that the rice transcription factor OsWRKY53, which is involved in a defense response, is also an important regulator of the BR signaling. OsWRKY53 positively regulates the BR signaling in rice, but also mediates a feedback repression of the BR biosynthetic genes. Interestingly, BRs stimulate the OsWRKY53 protein accumulation, but suppress the *OsWRKY53* gene transcription. Moreover, OsWRKY53 may attenuate its own expression. OsWRKY53 acts downstream of the OsBRI1 receptor and phosphorylation of OsWRKY53 by the OsMAPKK4 and OsMAPK6 kinases is crucial for its function [330].

## 6. Conclusions

Brassinosteroids regulate the broad range of processes during the plant life cycle. Research conducted for the last decades in Arabidopsis provided an insight into molecular mechanisms of the BR signaling and the BR-dependent gene expression. The BR signalosome is interconnected at various stages with signaling pathways of other phytohormones and environmental cues. This extensive and complex crosstalk allows the coordinated regulation of the physiological processes and rapid adaptation to environmental conditions. Nevertheless, further research is needed to fully clarify complicated regulatory mechanisms, which control the signaling interplay. Transfer of information about the crosstalk between the BR signalosome and other signaling pathways from the model species (Arabidopsis) to cereal crops is feasible, as the molecular mechanisms are evolutionarily conserved. It is an important issue as the BR signaling is considered as an ideal target to enhance crop yield and stress tolerance, especially taking into account the approaching global climate change.
